# Hybrid Cyclobutane/Proline-Containing Peptidomimetics: The Conformational Constraint Influences Their Cell-Penetration Ability

**DOI:** 10.3390/ijms22105092

**Published:** 2021-05-11

**Authors:** Ona Illa, Jimena Ospina, José-Emilio Sánchez-Aparicio, Ximena Pulido, María Ángeles Abengozar, Nerea Gaztelumendi, Daniel Carbajo, Carme Nogués, Luis Rivas, Jean-Didier Maréchal, Miriam Royo, Rosa M. Ortuño

**Affiliations:** 1 Departament de Química, Universitat Autònoma de Barcelona, 08193 Cerdanyola del Vallès, Spain; jimenabcn@gmail.com (J.O.); joseemilio.sanchez@uab.cat (J.-E.S.-A.); jeandidier.marechal@uab.cat (J.-D.M.); 2 Institut de Recerca Biomèdica, c/Baldiri Reixac 10, 08028 Barcelona, Spain; xpulido@ut.edu.co; 3 Centro de Investigación Biomédica en Red Bioingeniería, Biomateriales y Nanomedicina (CIBER-BBN), c/ Jordi Girona 18–26, 08034 Barcelona, Spain; 4 Departamento de Química, Universidad del Tolima, Santa Helena Parte Alta, Ibagué 730006299, Tolima, Colombia; 5 Centro de Investigaciones Biológicas Margarita Salas, c/ Ramiro de Maeztu 9, CSIC, 28040 Madrid, Spain; marabi66@hotmail.com (M.Á.A.); luis.rivas@cib.csic.es (L.R.); 6 Departament de Biologia Cellular, Fisiologia i Immunologia, Universitat Autònoma de Barcelona, 08193 Cerdanyola del Vallès, Spain; negazte@gmail.com (N.G.); carme.nogues@uab.cat (C.N.); 7 Institut de Química Avançada de Catalunya (IQAC-CSIC), c/ Jordi Girona, 18-26, 08034 Barcelona, Spain; daniel.carbajo@iqac.csic.es

**Keywords:** peptidomimetics, cell penetrating peptides, conformational bias, rigidity, charge-preorganization

## Abstract

A new family of hybrid β,γ-peptidomimetics consisting of a repetitive unit formed by a chiral cyclobutane-containing *trans*-β-amino acid plus a *N*^α^-functionalized *trans*-γ-amino-l-proline joined in alternation were synthesized and evaluated as cell penetrating peptides (CPP). They lack toxicity on the human tumoral cell line HeLa, with an almost negligible cell uptake. The dodecapeptide showed a substantial microbicidal activity on *Leishmania* parasites at 50 µM but with a modest intracellular accumulation. Their previously published γ,γ-homologues, with a cyclobutane γ-amino acid, showed a well-defined secondary structure with an average inter-guanidinium distance of 8–10 Å, a higher leishmanicidal activity as well as a significant intracellular accumulation. The presence of a very rigid cyclobutane β-amino acid in the peptide backbone precludes the acquisition of a defined conformation suitable for their cell uptake ability. Our results unveiled the preorganized charge-display as a relevant parameter, additional to the separation among the charged groups as previously described. The data herein reinforce the relevance of these descriptors in the design of CPPs with improved properties.

## 1. Introduction

Cell penetrating peptides (CPPs) [1,2,3] are potential carriers for drug delivery systems (DDS). They usually consist of peptide oligomers that convey the molecular payload into the intracellular space in the absence of a cognate transporter/receptor. One of their major drawbacks is their poor cellular specificity. This can be partially overcome by their local activation through the degradation of an inhibitory fragment carried out by specific proteinases, under environmental conditions (pH, hypoxia, etc.) or by the inclusion into their sequence of recognition motifs for a specific receptor located in the targeted cell [4]. A step ahead is the delivery of a cargo molecule into a certain organelle by inclusion of a particular import sequence or by physicochemical characteristics of a given peptide [5]. The pharmacological implementation of CPPs as DDS [6,7,8] is supported by their versatility to transport cargo molecules through covalent and non-covalent interactions, high bioavailability, fine tuning of their solubility, and by the feasibility to design multifunctional DDS [9]. As such, CPPs were implemented as a new tool in the therapeutics of a variety of diseases, with a special relevance in cancer [10].

CPP engineering aims to achieve an optimal biological performance by ensuring an adequate cell uptake and endosomal escape, with minimal toxicity, as well as to expand the half-life of the peptide under biological environments. This last property mostly relies on the improvement of its proteolytic resistance. To this end, the inclusion in their sequence of non-natural or stereochemically modified amino acids, as well as of peptide bond surrogates have been described [11].

The biological activity and selectivity of proteins and peptides is highly influenced by the conformational restriction of their polypeptide backbone. A case in point is the neuropeptide Y (NPY), which controls central and peripheral processes by the activation of the G protein coupled receptors Y_x_R. Meanwhile, the parental peptide shows promiscuous binding among different receptors of this family, analogues of its *C*-terminal fragment containing cyclobutane or cyclopentane β-amino acids displayed exclusively binding to Y_4_R [12]. A brand-new example is the incorporation of six proline residues in the spike glycoprotein of coronavirus virions with improved recombinant expression, and tolerance heat stress, thus allowing their storage at room temperature [13]. Otherwise, local constriction in biologically active peptides is another useful tool to improve their activity. For instance, the modification of the hydrazide bridge in biphalin, an opioid octapeptide, significantly enhances its analgesic properties [14]. This is the case, for instance, of biphalin analog AM94, where the native hydrazine linker has been replaced by a more constrained and less planar piperazine ring. AM94 displayed a higher potency than biphalin and a comparable effect with morphine [15]. Another example is provided by the incorporation of side-chain constrained α- or β-substituted amino acids into bioactive peptide ligands as key pharmacophore residues. It has proven to be a powerful method for understanding ligand–receptors binding interaction and in peptidomimetics design [16].

Conformational constraints were also introduced to stabilize the secondary structure of CPPs, sometimes associated with a better cell uptake [11]. For cationic CPPs, uptake and toxicity are highly dependent on the number and spatial distribution of positive charges throughout their sequence. With these two goals in mind, diverse CPPs were designed by combining in a single unit two cyclic amino acids, mainly proline and γ-aminoproline [17,18], helical peptide foldamers [19,20,21], and cyclic peptide backbones [21,22,23,24].

**Figure 1:**
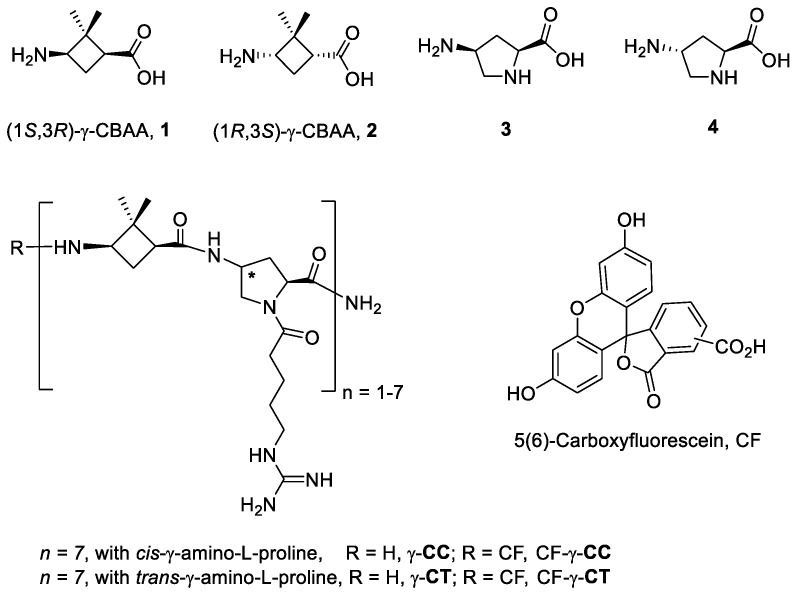
Monomers, type of peptides, and carboxyfluorescein (CF) conjugates investigated in our earlier studies [26,27,29].

In previous works, we reported the biological performance as CPP of hybrid γ,γ-peptides formed by repetition of a dimeric unit constituted by a protected derivative of either cyclobutane γ-amino acids (γ-CBAA), **1** or **2** combined with *cis*-γ-amino-l-proline, **3** (Figure 1). High-resolution NMR spectra of these compounds showed very rigid and compact structures due to the intra- and inter-residue hydrogen-bonded ring formation [26].

For these peptides, the uptake by tumoral human cell line HeLa increased with its length, while the stereochemistry of the γ-CBAA was scarcely relevant. A good polar-hydrophobicity balance was achieved by the alternation of the guanidinium groups and the hydrophobicity of the (*gem*-dimethyl) cyclobutane ring [26,27]. These cyclobutane-containing CPPs showed a lower toxicity than those made exclusively of guanidinylated γ-amino proline residues though preserving a similar cell uptake [28], likely due to their halved number of guanidinium groups compared with the γ-aminoproline peptides of the same length.

Later, we synthesized the oligomers (from 8 to 14 residues) formed by **1** and *cis*- or *trans*-γ-amino-l-proline, **3** and **4**, as the bases for γ-**CC** and γ-**CT**, respectively (Figure 1). These peptides bear guanidinium groups attached to the backbone through a flexible spacer. They were conjugated to carboxyfluorescein (CF) used as a fluorophore and were evaluated as CPPs and as selective vectors for anti-*Leishmania* DDS. These peptides and their respective CF-conjugates lacked cytotoxicity on HeLa cells with a moderate cell-uptake on them. In contrast, both γ-**CC** and γ-**CT** tetradecamers were microbicidal on *Leishmania* beyond 25 µM, with a significant intracellular accumulation in this protozoan parasite [29].

**Figure 2:**
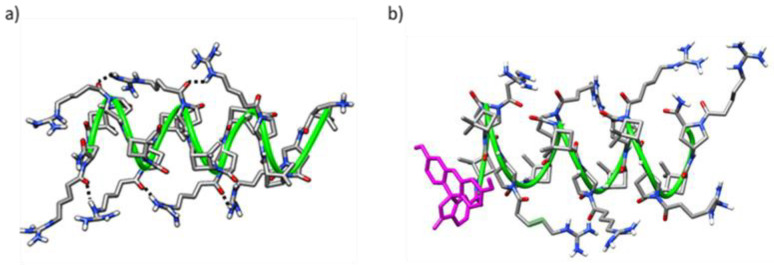
Representative MD conformations for (**a**) peptide γ-**CT**, with inter-residue interactions are marked; and (**b**) CF-γ-**CT**. The green ribbon stands out for the peptide scaffold. CF moiety is represented in magenta.

These peptides adopted a defined conformational preference in solution, as assessed by circular dichroism (CD) spectroscopy. In addition, well-defined conformations for both tetradecamers were inferred by molecular dynamics (MD) simulations, driven by the formation of intra- and long-range inter-residue hydrogen bonds. As an example, representative conformations for γ-**CT** and for CF-γ-**CT** are displayed in Figure 2. To note, the conformational restrictions for γ,γ-peptides are much higher than for peptides made of α-amino acids. Moreover, an important fact is the location of polar guanidinium groups at the periphery of the peptide facing the solvent. This will improve their solubility and the interaction with the negatively charged peptidoglycans of the extracellular matrix of the targeted cell [29].

**Figure 3:**
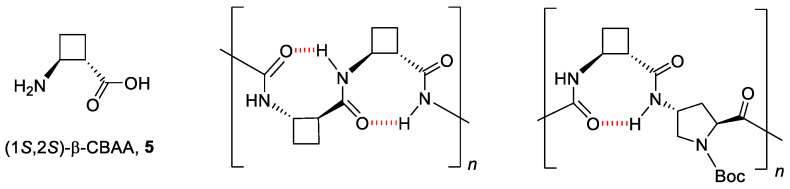
Eight-membered hydrogen-bonded rings in oligomers containing the cyclobutane amino acid **5.**

To pursue a better definition on the role of conformational restriction of cyclobutane-containing peptidomimetics with respect to their cell penetrating activity, we synthetized new hybrid β,γ-oligomers that incorporate the highly constrained (1*S*,2*S*)-2-aminocyclobutane-1-carboxylic acid, **5**, (Figure 3). In previous studies, it was observed that the inclusion of **5** in short oligomers (2–12 residues) led to well defined helical folding in solution prompted by the formation of eight-membered hydrogen-bonded rings involving *i* and *i* + 1 residues [30,31,32]. This type of hydrogen bonding was also found in preliminary studies on hybrid β,γ-oligomers (2–4 residues) containing *N*^α^-Boc aminoproline derivatives (Boc = *tert*-butoxycarbonyl) [33] (Figure 3).

**Figure 4:**
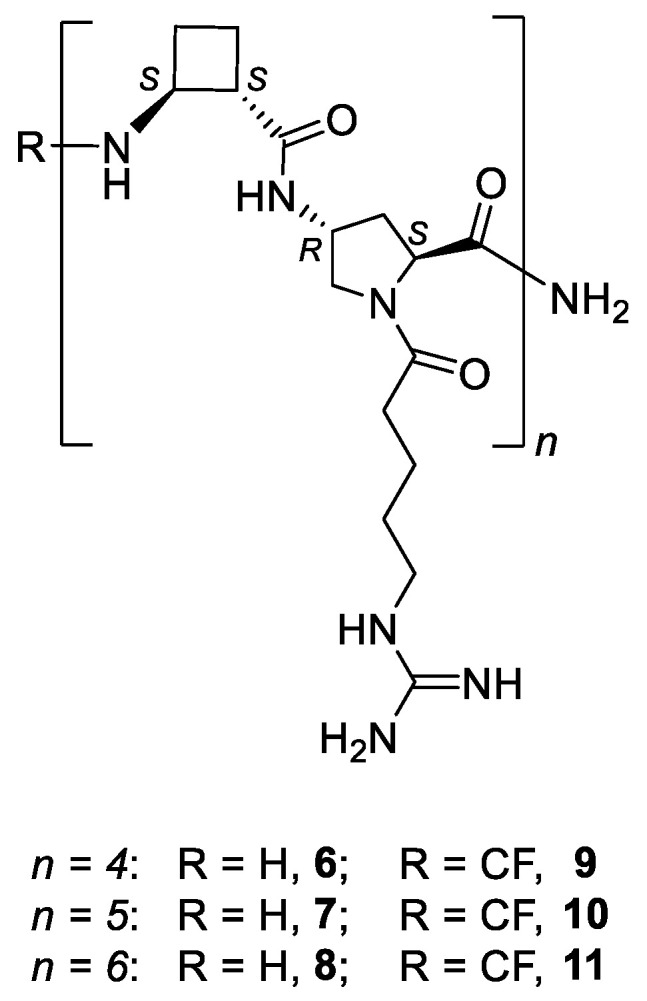
Hybrid β,γ-peptides and their CF-conjugates involved in this work.

In order to get a better insight into the relevance of conformational constraints in the CPP scaffolds and their biological performance, peptides **6**–**11** (Figure 4) were compared with the homonymous oligomers containing the γ-amino acid **1** (Figure 1). Their cell penetrating activity was assayed in two different biological models, HeLa cells and the two major forms of *Leishmania*, a human protozoan parasite. In addition, MD simulations were carried out to predict the representative conformations. Altogether, the importance of a specific topology of the guanidium group on CPPs incorporating β,γ- or γ,γ- amino acids was verified.

## 2. Results and Discussion

### 2.1. Synthesis of the Peptides

To analyze the role of peptide length on the cell uptake, a series of hybrid β,γ-peptides **6–11** (Figure 4, *n* = 4–6) was synthesized. They were formed by the repetition of a dipeptide motif made up by (1*S*,2*S*)-β-CBAA, **5** [34] and *trans*-γ-amino-l-proline, **4**. All of them were prepared using standard protocols of solid phase synthesis methods (SPPS), either in their free *N*-terminus form (**6**–**8**) or with CF (**9**–**11**), as detailed in Section 4 and in the Supplementary Materials (Supplementary Tables S1–S3).

TAT_48–57_ (transactivator of transcription, TAT) [35,36,37] was also synthesized as a reference CPP, either with a free or carboxyfluoresceinated *N*-terminus as previously described [29].

### 2.2. Cytotoxicity and Cellular Uptake in HeLa Cells

**Figure 5:**
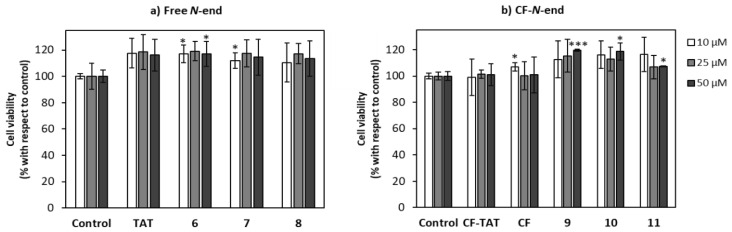
Cytotoxicity of peptides **6**–**11** on HeLa cells. Peptides with (**a**) Free terminal amino group; (**b**) terminal amino group conjugated with CF. Cells were incubated with the respective peptide for 24 h and their viability assayed by MTT reduction. Cell viability was expressed as the percentage of MTT reduction with respect to control cells ± SD. Experiments were repeated thrice independently. Statistical significance respect to untreated control (*p* ≤ 0.05, (*); *p* ≤ 0.001, (***)).

The cellular viability of peptides **6**–**11** was determined by the reduction of MTT (3-(4,5-dimethylthiazol-2-yl)-2,5-diphenyltetrazolium bromide) assay [38] after 24 h of cell incubation with the respective oligomer. The measure of the formazan formed from the reduction of MTT by mitochondrial dehydrogenases is directly related to the cellular viability (Figure 5). Even at the highest concentration assayed (50 µM), viability was over 90% for this set of peptides. Peptide toxicity was not dependent either on the number of guanidinium groups in the sequence or on the carboxyfluoresceination of the terminal amino group, in agreement with the previous data on their γ,γ-counterparts, γ-CC and γ-CT (Figure 1) [29].

**Figure 6:**
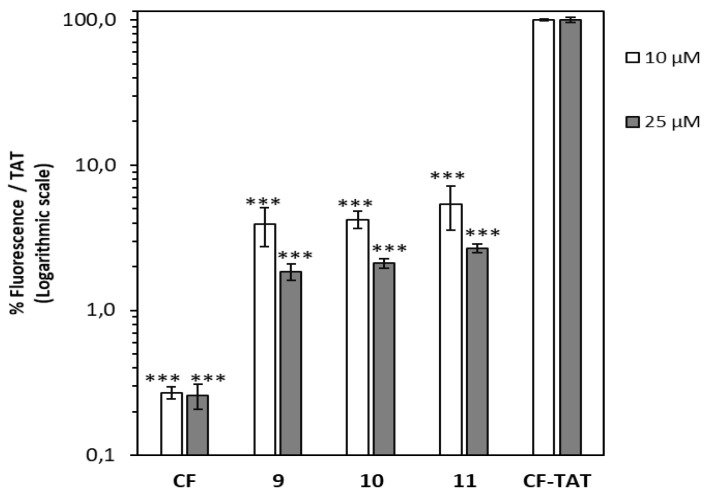
Cellular internalization of carboxyfluoresceinated peptides **9**–**11** normalized with respect to CF-TAT (100%). HeLa cells were incubated with the respective peptide at 10 μM (empty column), or 25 μM (black column) for 2 h at 37 °C, and the level of fluorescence associated with the cells assessed by flow cytometry (λ_EXC_ = 488 nm and λ_EM_ = 530 nm). Results were expressed as mean ± SD. Three independent experiments were carried out. Statistical significance respect to CF-TAT (*p* ≤ 0.001, (***)).

Their cell internalization ability in HeLa cells was assessed by flow cytometry. To this end, the cell-associated fluorescence was quantified after incubation with peptides **9**–**11** at 10 and 25 µM and compared with that obtained with CF-TAT as a standard reference (Figure 6). Results showed a modest cell-uptake for all peptides, being the dodecamer the peptide with the highest value.

**Figure 7:**
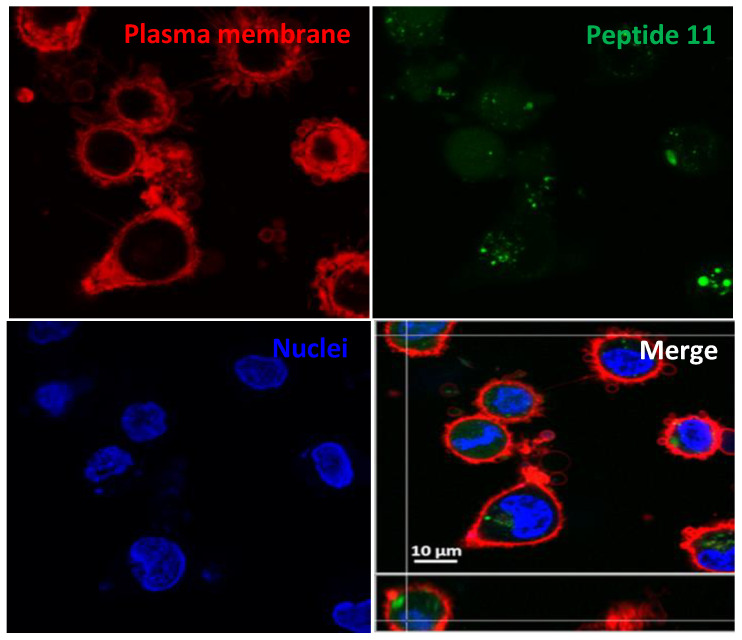
Intracellular location of the fluorescent peptide **11.** Confocal microscopy of HeLa cells incubated with the carboxyfluoresceinated dodecamer **11** (incubation conditions: 2 h, 37 °C, 10 µM) (green fluorescence λ_EXC_ = 488 nm; λ_EM_ = 510 nm). Plasma membrane was additionally stained with CellMask, deep red (red fluorescence, λ_EXC_ = 658 nm; λ_EM_ = 690 nm), and nucleic acids with Hoechst (blue fluorescence, λ_EXC_ = 405 nm; λ_EM_ = 460 nm). Magnification bar: 10 µm.

The intracellular uptake of the peptides was assessed by confocal microscopy. Figure 7 shows HeLa cells after 24 h incubation with the dodecamer **11** at 10 µM. CellMask deep red and Hoechst were used to stain the plasma membrane (red), and DNA (blue), respectively. The CF-peptide accounts for the green fluorescence.

### 2.3. Uptake, Microbicidal Activity, and Intracellular Location of Peptides on Leishmania Parasites

Once the lack of cytotoxicity and poor uptake of the peptides respect to TAT on HeLa cells was evidenced, their performance on Leishmania parasites was approached. This is a challenging human protozoan parasite in reference to peptide membrane interaction. The cell membrane of *Leishmania* is reinforced by a longitudinal subpellicular layer of microtubules [39]. This confines all the membrane traffic to a small membrane area devoid of microtubules, the flagellar pocket [40]. In addition, it is endowed with a strong proteolytic armamentarium not only in the surface, but also in lysosomal enzymes, especially in the amastigote form of those species, as *L. pifanoi*, belonging to the *mexicana* complex [41,42]. To this end, peptides were assayed for viability, uptake and location on the protozoan *Leishmania* parasites.

The decrease on parasite viability was dependent both on the length of the peptide and on its concentration. The toxicity increases with the length of the peptide for both forms of the parasite, the dodecamer (**11**) and the decamer (**10**) being substantially more toxic than the octamer (**9**), likely due to the increase of the overall positive charge of the peptide [43]. This inhibitory effect was lower for *L. pifanoi* amastigotes, despite of their high content in cysteine proteinases with a broad substrate specificity, accounting for 5% of their total protein content. A similar trend was also observed for their γ, γ-counterparts, although with a higher quantitative effect [29].

Because of the nil inclusion of α-amino acids in the peptide composition, the divergence among *Leishmania* stages is not due to differences in their proteinase content, but likely mirrored differences in their membrane architecture. Almost 40% of the promastigote surface is covered with lipophosphoglycan formed by repetition of a phosphorylated disaccharide unit, whereas the amastigote hardly expresses a few hundred copies [44]. This oligosaccharide forms a strong anionic coating that likely acts as an initial anchor point for the peptides prior to their internalization.

**Figure 8:**
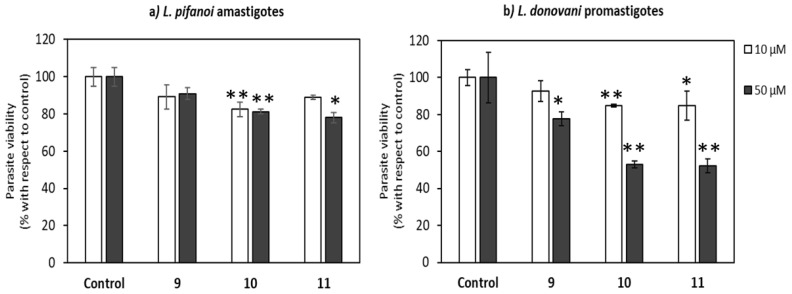
Leishmanicidal effect of the peptides. Panel (**a**) Viability of *L. pifanoi* amastigotes. Panel (**b**) *L. donovani* promastigotes. Viability was measured after incubation with peptides **9**–**11** for 4 h. *Leishmania* parasites (20 × 10^6^ cells/mL) were incubated with the peptides, and MTT reduction measured immediately after 4 h incubation at 26 °C at 10 (white column) or 50 µM (black column) final peptide concentration. Viability was represented as the percentage of MTT reduction (mean ± SD). Samples were made in triplicate. Statistical significance respect to untreated control (*p* ≤ 0.05, (*); *p* ≤ 0.01, (**)).

**Figure 9:**
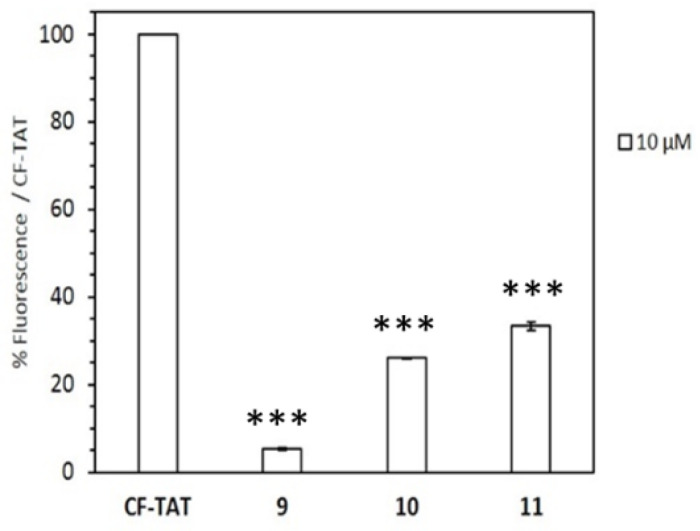
Uptake of CF-β,γ-peptides **9–11** by *Leishmania donovani* promastigotes. Parasites (20 × 10^6^ cells/mL) were incubated for 4 h with the respective peptides at 10 μM. Afterwards, peptide incorporation into parasites was assessed by flow cytometry (λ_EXC_ = 488 nm, λ_EM_ = 519 nm) and represented as the percentage with respect to CF-TAT ± SD. Error bars represent standard deviation (SD) from the mean value of three independent experiments with each peptide. Samples were made in triplicate. Statistical significance respect to untreated control. (*p* ≤ 0.001, (***)).

To ascertain the role of length of the peptide in its uptake on *L. donovani* promastigotes, the set of β,γ-peptides were incubated for 4 h at a fixed concentration of 10 μM. The choice of these conditions relies both on the higher differences in cytotoxicity observed for promastigotes over amastigotes, as well as to the scarce toxicity of the peptides at this concentration, always less than <20% (Figure 8). According to Figure 9, the peptide uptake increased with its length although for all the β,γ-peptides, the uptake was consistently lower than that obtained for CF-TAT at identical concentration, used as a reference for CPP.

**Figure 10:**
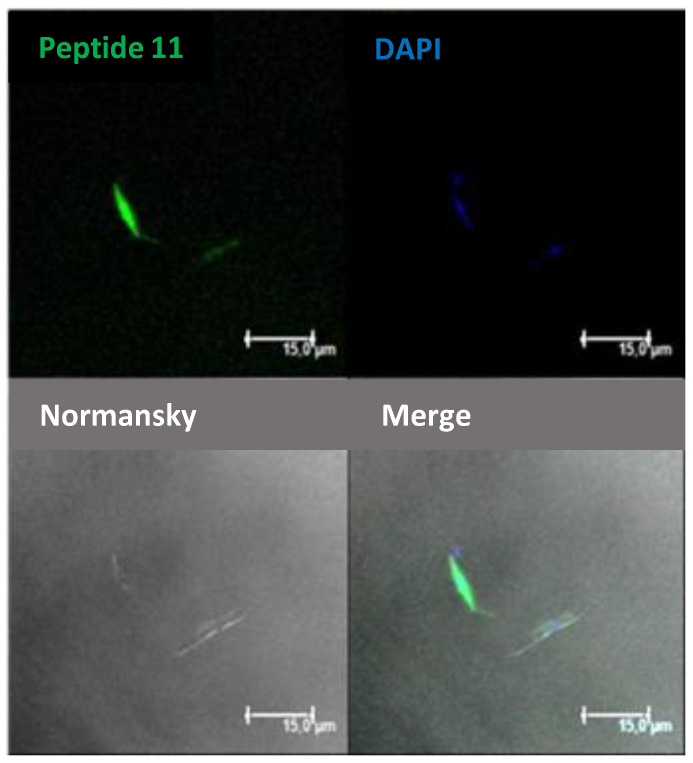
Confocal microscopy of *Leishmania donovani* promastigotes incubated with dodecamer **11** (10 μM final concentration, 4 h, 26 °C). Afterwards, they were observed without further fixation. CF-peptides (green fluorescence: λ_EXC_ = 488 nm/λ_EM_ = 519 nm). DAPI (DNA probe, blue fluorescence: λ_EXC_ = 358 nm/λ_EM_ = 461 nm). Magnification bar = 15 μm.

The internalization of these peptides in *Leishmania donovani* promastigotes was assessed by confocal microscopy. Thus, promastigotes were incubated with peptides **9–11** as well as TAT at a final concentration of 10 μM for 4 h at 26 °C. DAPI was used as a nucleic acid dye to stain the nucleus and the kinetoplast (blue fluorescence) (Figure 10).

### 2.4. Molecular Modeling

**Figure 11:**
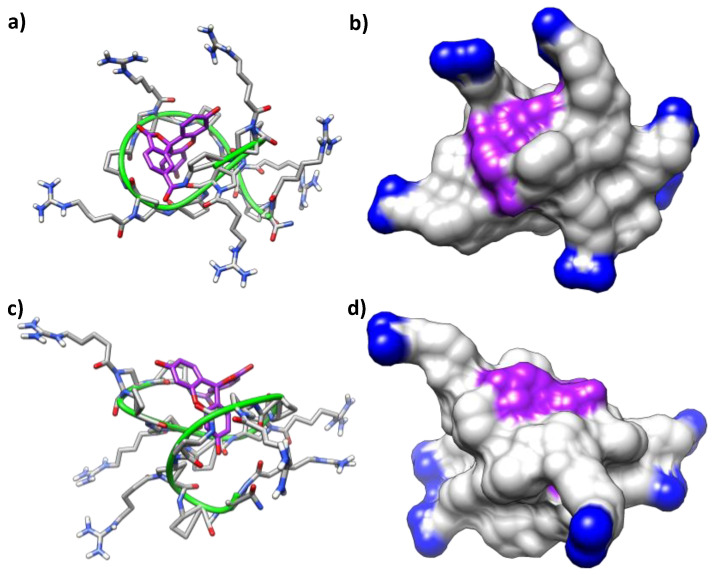
MD simulation of the CF-conjugate **11.** Representative snapshots of the two most populated clusters for the peptide are included. Ribbon and stick representation (**a**,**c**), with the CF motif highlighted in purple. Surface representation (**b**,**d**), with the CF motif highlighted in purple and the terminal nitrogen atoms of the guanidinium groups highlighted in blue. (**a**,**b**) Representative structure of cluster one. (**c**,**d**) Representative structure of cluster two.

To ascertain the folding of the peptides in aqueous solution as well as the influence of the fluorophore moiety (CF), molecular dynamics (MD) simulations under an explicit solvent scenario were carried out on the hybrid β,γ-peptide **8** and its CF-conjugate **11** (MD length of 900 ns and 600 ns, respectively). Both oligomers showed highly disorganized structures along the MD simulations, without any ordered secondary structure motif such as helical or β-sheet-like structures. Considering the backbone carbons of the peptides and a RMSD threshold of 2.0 Å, no cluster accounting for at least 1% of the simulation was found for the peptide **8**, whereas the simulation of the CF-conjugate **11** led to twenty clusters containing, at least, 1% of the frames. Consequently, the incorporation of the CF induced a slight structural stabilization of the peptide. An inspection of the representative structures of the two most populated clusters (21% and 8% of the frames) supported the acquisition by the peptide of a globular shape where the peptide wrapped the CF moiety (Figure 11). This trend is observed in a series of MD clusters, but its high dynamics precluded a better identification of any stable interactions between the CF and the peptide.

As mentioned in the introduction, previous studies reported well-defined secondary structures for short oligomers made up exclusively with (1*S*,2*S*)-2-aminocyclobutane-1-carboxylic acid, **5,** driven by the formation of eight-membered hydrogen-bonded rings (Figure 3) [30,31,32,33]. Strikingly, no stable hydrogen bonding patterns were found along the MD simulations, with a total of 721 and 456 different hydrogen bonds observed that appeared/disappeared throughout the trajectories of the peptide **8** and the CF-conjugate **11**, respectively. None of them stands more than 15% of the time of simulation, suggesting that eight-membered stable rings are either absent, or their formation is quite transitory, precluding their observation. Another relevant factor involved in the cellular uptake of CPPs is the cationic character of the peptide, as it endorses a high affinity binding into the anionic proteoglycans exposed at the cell-surface, mandatory for their further internalization and endosomal escape [45,46,47]. A proper orientation of the cationic groups facing the solvent will speed up and strengthen this interaction. In this regard, most of the time the terminal nitrogen atoms of the guanidinium groups are exposed to the external aqueous medium according to MD simulations for peptide **8** and its CF-conjugate **11**.

Apart from visual inspection of the representative structures (Figure 11), a solvent accessible surface area (SASA) analysis confirmed that the guanidinium groups of oligomers **8** and **11** have a good orientation toward the solvent, with average SASA values of 1.145 ± 0.084 nm^2^ and 1.160 ± 0.078 nm^2^, respectively (Table 1).

Otherwise, the influence of the spatial arrangement of positively charged amino acids of CPPs on their translocation profile is still controversial. First, a study on a series of arginine-rich CPPs [23] inferred that large inter-guanidinium distances, up to 15 Å, enhance uptake kinetics. However, a subsequent study suggested that shorter distances from 8 to 10 Å were necessary to match the topology of anionic groups in cell-surface glycans [20]. Indeed, despite the conformational flexibility of the lipophosphoglycan of *Leishmania donovani*, as a putative anchor for CPPs, a length of 160 Å was described for a structure formed by the extended conformation of the 16mer of the phosphorylated disaccharide, its repetitive forming unit. This accounts for an average separation of 10 Å [48].

In a recent study of ours on γ,γ-CPPs, MD simulations suggested inter-guanidinium distances in the range 8–10 Å both for the most active peptides (Figure 2) or their CF-conjugates [29]. Nevertheless, a similar analysis on **8** and **11** shows average inter-guanidium distances of 14.84 ± 4.46 Å and 16.42 ± 4.17 Å, respectively, not matching the reported as optimal distance among close guanidium groups. These observations offer a possible reason for the poorer activity of the current β,γ-CPPs since they are not prone to form the suitable charge distribution for efficient cell membrane translocation.

## 3. Conclusions

A new family of octa-, deca-, and dodecameric hybrid β,γ-peptides and their CF-conjugates were synthesized and evaluated as CPPs for toxicity and cell uptake ability. These peptides were made by a repetitive unit made of a chiral cyclobutane-containing *trans*-β-amino acid linked to a *N*^α^-functionalized *trans*-γ-amino-l-proline. Their uptake and cytotoxicity on HeLa cells were not significant. In contrast, toxicity on *Leishmania pifanoi* amastigotes and especially on *Leishmania donovani* promastigotes was remarkable at 50 µM, with a toxicity ranking: 12-mer ≅ 10-mer >8-mer. Peptide internalization by *Leishmania* was practically nil on *L. pifanoi* amastigotes and modest on *L. donovani* promastigotes with a substantial increase with the length of the peptide. By MD simulations, dodecamer **8** and its CF-conjugate **11** are devoid of any conformational bias. These results differ from those reported for their γ,γ-homologues, with a cyclobutane-containing *cis*-γ-amino acid and a *cis*- or *trans*-γ-amino-l-proline as their structural units [29]. This γ,γ-CPP family adopted a well-defined conformation in solution, with modest uptake, and a toxicity on *Leishmania* parasites higher than the hybrid β, γ-peptides described herein.

The SASA analysis of the β,γ-dodecamers (**8**, and its CF-version, **11**) confirmed that the guanidinium groups are exposed to the external aqueous medium for most of the time with an average inter-guanidinium distance of about 15 and 16 Å, respectively. This value did not fall into the interval 8–10 Å described as the optimal distance to match with the anionic groups of cell-surface glycans [20,48]. On the contrary, the γ,γ-counterparts, with a less constrained 1,3-disubstituted cyclobutane moiety, presented more stable conformations with inter-guanidium distances of 8–10 Å for the most active CPPs. Consequently, our results extended the notion of conformational restraint, previously described for α-peptides [49], to lineal CPPs by the inclusion of non-natural amino acids endowed with limited conformational freedom into their sequence. As a conclusion, the use of peptides with tuned conformational restriction, achieved by a rational incorporation of cyclobutane β- or γ-amino acids in their sequences, allows the exploration of the topology of guanidium groups and their intramolecular dynamics with a mobility limited by the stiffness of the peptide skeleton. Consequently, both stable peptide folding and convenient guanidinium structural patterns are reinforced as CPP key descriptors for an efficient cell-membrane translocation. As such, considering dynamical simulations as a part of future CPPs-engineering protocols appears promising.

## 4. Materials and Methods

The reagents and solvents were purchased from commercial sources and used without further purification. Unless otherwise stated, the reagents were purchased from Sigma Aldrich, Barcelona, Spain.

### 4.1. Synthesis of the β, γ-Peptides 6–11 and TAT_48–57_ Peptide

β,γ-Peptides were synthesized manually on solid-phase using Aminomethyl-ChemMatrix^®^ resin (f = 0.74 mmol NH_2_/g resin). The resin was conditioned with successive washes with DCM (dichloromethane), TFA (trifluoroacetic acid)/DCM (4:5, *v/v*), DIPEA (*N*-diisopropylethylamine)/DCM (4:5, *v/v*), DMF (dimethylformamide) and DCM. The linker was coupled using 3:3:3 Fmoc-Rink amide-Linker/DIC/OxymaPure^®^ (Fmoc = fluorenylmethoxycarbonyl, DIC = *N,N′*-Diisopropylcarbodiimide) in DMF for 1 h. β,γ-Peptides **6**-**11** backbone was elongated by alternate addition of (2*S*,4*S*)-4-Fmoc-amino-1-Alloc-pyrrolidin carboxylic acid (Alloc = Allyloxycarbonyl) and (1*S*,2*S*)-2-Fmoc-aminocyclobutane-1-carboxylic acid using DIC/OxymaPure^®^ (3 equiv/3 equiv/3 equiv) as coupling reagents in DMF for 2 h. Fmoc group was released by treatment with piperidine-DMF (2:8, *v/v*). After each Fmoc elimination step, the peptidyl–resins were washed with DMF and DCM. The couplings were monitored by the Kaiser (ninhydrin) test. Once the peptide backbone syntheses were finished, Alloc groups were eliminated by catalytic reduction using 12/0.1 PhSiH_3_/Pd(PPh_3_)_4_ in DCM, and the guanidinium-containing side-chain introduced to the α-amine function of the Amp residues (Amp = 4-aminoproline). Then, the Fmoc group in *N*-terminal position was eliminated. At this point, the peptidyl-resin was split in two parts. One of these two fractions was conjugated to 5(6)-Carboxyfluorescein using CF/OxymaPure^®^/PyBOP/DIPEA (4:6:4:6) in DMF (PyBOP = benzotriazol-1-yloxytripyrrolidinophosphonium hexafluorophosphate). Finally, β, γ-peptides **9**–**11** were cleaved from the peptidyl-resins by treatment with TFA/H_2_O/TIS (95:2.5:2.5, *v/v/v*) (TIS = triisopropylsilane) for 3 h, precipitated in cold diethyl ether, dissolved in CH_3_CN/H_2_O (1:1, *v/v*) and lyophilized. The synthesis of TAT_48–57_ was performed by using standard Fmoc/*tert*-Bu SPPS methods as previously described [29]. See the Supplementary Materials for more details and Supplementary Tables S1–S3.

### 4.2. Peptide Purification

Crude peptides were purified by semipreparative RP-HPLC-UV-MS using a system composed by a binary gradient Waters 2545, a Waters Alliance 2767 sample manager module and an automatic fraction collector coupled to Waters 2487 dual UV-vis absorbance detector and an electrospray ion source (ESI-MS) Micromass ZQ mass spectrometer detector. The chromatographic separation of the peptides was achieved using a semipreparative column XBridge^®^ Prep BEH C_18_ (19 × 100 mm, 5 µm) (Waters, Cerdanyola del Vallès, Spain) with dual solvent system formed by A: 0.1% TFA in H_2_O and B: 0.1% TFA in CH_3_CN at a flow rate of 25 mL/min. λ = 220 nm. Elution gradient was specific for each peptide.

### 4.3. Peptide Characterization

Peptide analysis and determination of the respective retention time (t_R_), analytical column XBridge™ BEH 130 C18 (4.6 × 100 mm, 3.5 μM) (Waters, Cerdanyola del Vallès, Spain) was used. Peptide elution was monitored at 220 nm.

#### 4.3.1. Octameric Peptide (6)

Purification: RP-HPLC-UV-MS gradient: 5 to 40% B in 10 min; 40 to 60% B in 1 min, and to 100% B in 2 min. Characterization: RP-HPLC. Purity >95%, t_R_ 3.4 min. *m/z* (ESI): Ms Calcd for C_64_H_107_N_25_O_12_ [M + 3H/3]^+^: 474.6; Experimental: 473.2. Ms Calcd para C_64_H_107_N_25_O_12_ [(M + 4H)/4]^+^: 355.6; Experimental: 355.7. Ms Calcd for C_64_H_107_N_25_O_12_ [(M + 5H)/5]^+^: 284.7; Experimental: 284.7. MALDI-TOF: Ms Calcd for C_64_H_107_N_25_O_12_ [(M + H)]^+^: 1419.72; Experimental: 1419.02. Ms Calcd para C_64_H_107_N_25_O_12_ [M + Na]^+^: 1457.82; Experimental: 1457.00.

#### 4.3.2. Decameric Peptide (7)

Purification: RP-HPLC-UV-MS gradient: 5 to 40% B in 10 min; 40 to 60% B in 1 min, and to 100% B in 2 min. Characterization: RP-HPLC. Purity >98%, t_R_ 3.4 min. *m/z* (ESI): Ms Calcd for C_80_H_133_N_31_O_15_ [(M + 3H)/3]^+^: 590.7; Experimental: 590.7. Ms Calcd for C_80_H_133_N_31_O_15_ [(M + 4H)/4]^+^: 445.2; Experimental: 445.3. Ms Calcd for C_80_H_133_N_31_O_15_ [(M + 5H)/5]^+^: 354.8; Experimental: 354.7. MALDI-TOF: Ms Calcd for C_80_H_133_N_31_O_15_ [M + H]^+^: 1769.06; Experimental: 1769.22.

#### 4.3.3. Dodecameric Peptide (8)

Purification: RP-HPLC-UV-MS gradient: 10 to 60% B in 11 min, and to 100% B in 2 min. Characterization: RP-HPLC. Purity >99%, t_R_ 3.5 min. *m/z* (ESI): Ms Calcd for C_96_H_159_N_37_O_18_ [(M + 4H)/4]^+^: 530.8; Experimental: 530.9. Ms Calcd for C_96_H_159_N_37_O_18_ [(M + 5H)/5] ^+^: 424.9; Experimental: 424.8. MALDI-TOF: Ms calcd for C_96_H_159_N_37_O_18_ [M + H]^+^: 2119.27; Experimental: 2119.47. Ms Calcd for C_96_H_159_N_37_O_18_ [M + Na]^+^: 2141.26; Experimental: 2141.40.

#### 4.3.4. Octameric CF-Peptide (9)

Purification: RP-HPLC-UV-MS gradient: 5 to 40% B in 10 min; 40 to 60% B in 1 min, and to 100% B in 2 min. Characterization: RP-HPLC. Purity >99%, t_R_ 5.9 min. *m/z* (ESI): Ms Calcd for C_85_H_117_N_25_O_18_ [(M + 2H)/2]^+^: 889.5; Experimental: 889.3. Ms Calcd for C_85_H_117_N_25_O_18_ [(M + 3H)/3]^+^: 593.3; Experimental: 593.7. Ms Calcd para C_85_H_117_N_25_O_18_ [(M + 4H)/4]^+^: 445.2; Experimental: 445.1. Ms Calcd for C_85_H_117_N_25_O_18_ [(M + 5H)/5]^+^: 356.4; Experimental: 356.3. MALDI-TOF: Ms calcd for C_85_H_117_N_25_O_18_ [(M + 3H)]^+^: 1778.03; Experimental: 1778.20. Ms Calcd for C_85_H_117_N_25_O_18_ [M + Na]^+^: 1800.02; Experimental: 1799.22.

#### 4.3.5. Decameric CF-Peptide (10)

Purification: RP-HPLC-UV-MS gradient: 5 to 50% B in 10 min; 50 to 60% B in 1 min, and to 100% B in 2 min. Characterization: RP-HPLC. Purity >99%, t_R_ 5.5 min. *m/z* (ESI): Ms Calcd for C_101_H_143_N_31_O_21_ [(M + 3H)/3]^+^: 710.1; Experimental: 710.4. Ms Calcd for C_101_H_143_N_31_O_21_ [(M + 4H)/4]^+^: 532.8; Experimental: 532.8. Ms Calcd for C_101_H_143_N_31_O_21_ [(M + 5H)/5]^+^: 426.4; Experimental: 426.4. MALDI-TOF: Ms Calcd for C_101_H_143_N_31_O_21_ [M + H]^+^: 2127.11; Experimental: 2127.32.

#### 4.3.6. Dodecameric CF-Peptide (11)

Purification: RP-HPLC-UV-MS gradient: 10 to 60% B in 11 min, and to 100% B in 2 min. Characterization: RP-HPLC. Purity >99%, t_R_ 4.8 min. *m/z* (ESI): Ms Calcd for C_117_H_169_N_37_O_24_ [(M + 3H)/3]^+^: 826.9; Experimental: 826.5. Ms Calcd for C_117_H_169_N_37_O_24_ [(M + 4H)/4]^+^: 620.4; Experimental: 620.3. Ms Calcd for C_117_H_169_N_37_O_24_ [(M + 5H)/5]^+^: 496.5; Experimental: 496.5. MALDI-TOF: Ms Calcd for C_117_H_169_N_37_O_24_ [M + H]^+^: 2477.31; Experimental: 2477.46.

### 4.4. Cellular Viability, Internalization, and Localization Experiments with HeLa Cells

The HeLa cell line, derived from a human cervical cancer, was used to perform the biological assays. CPP dissolved in non-supplemented Minimum Essential Medium (MEM) (Gibco, Thermo Fisher Scientific, Cornellà de Llobregat, Spain) at their final concentration were sterilized by filtration through a Whatman^®^ Puradisc 0.2 µm polycarbonate filter.

#### 4.4.1. HeLa Cells Culture

The cells were cultured in 25 cm^2^ flasks in MEM culture media supplemented with 10% of heat inactivated fetal bovine serum (FBS, Gibco) plus 2 mM L-glutamine (Biowest, Labclinics, Barcelona, Spain) at 37 °C, saturated humidity and 5% of CO_2_ (standard conditions).

#### 4.4.2. Cellular Viability

The MTT assay was used to assess the cytotoxicity of the peptides. This method is based on the ability of living cells to reduce the MTT to formazan salts by mitochondrial reductases. HeLa cells were seeded into 24 microwell plates at 6 × 10^4^ cells/mL (30,000 cells/well). After 24 h of incubation, the medium was replaced with fresh medium with the respective peptide concentration and incubated for 24 h. Then, the cells were washed three times with Hanks buffered saline solution (HBSS) (Biowest, Labclinics, Barcelona, Spain), and 500 µL of 0.1 mg/mL MTT in HBSS were added to the cell suspension and incubated for 3 h at 37 °C in darkness. The cell layer was dried in darkness, and the resulting formazan solubilized in pure DMSO. The absorbance was measured at 540 nm in an X3 Multilabel Plate Reader coupled to Perkin Elmer 2030 Manager control software. At least three independent experiments with four different replicates of each peptide and concentration were performed. Controls, non-treated cells, or cells incubated with CF were included at identical concentrations. The absorbance of non-treated cells was taken as 100% cellular viability.

#### 4.4.3. Peptide Internalization

For flow cytometry experiments, HeLa cells were seeded into 35 mm culture dishes (2 × 10^5^ cells/dish). After 24 h incubation under standard conditions, culture medium was removed, and cells were incubated for 2 h with the corresponding peptides at 10 and 25 μM. Next, the culture medium was removed, the cells were washed twice with HBSS and then trypsinized with 0.5 mL of 0.25% trypsin-EDTA (Gibco, Thermo Fisher Scientific, Cornellà de Llobregat, Spain). After 5 min incubation at 37 °C, 2 mL of MEM + 10% FCS was added to the cells to stop trypsinization and the mixture was centrifuged (5 min, 300× *g*). The cells were then additionally washed with 2 mL of HBSS under the same conditions. Finally, the cell pellet was resuspended in 200 μL of PBS at pH = 6.0 to detach any peptide adhering to the plasma membrane. To exclude dead cells from gating, 5 µg/mL propidium iodide (PI, Sigma-Aldrich, Barcelona, Spain) was added to the cells immediately before the flow cytometric analysis, carried out in a BD FACSCanto cytometer (Bio-Rad, Alcobendas, Spain) coupled to FACSDiva v.7.0 software using 488 nm and 635 nm lasers to excite the peptides and PI, respectively.

A total of 10,000 single cells were analyzed per sample, and at least three independent experiments were performed with each peptide and concentration. Untreated cells (autofluorescence control) and cell cultures incubated with TAT as positive reference CPP, and CF as negative reference, were included in each experiment. The fluorescence intensity of cells treated with CF was taken as the arbitrary unit for normalization. For confocal microscopy, HeLa cells were seeded into glass bottom culture dishes (MatTek, Bratislava, Slovakia) at a density of 2 × 10^5^ cell/dish. After 24 h of incubation, the culture medium was removed, and the cells were incubated for 2 h in the presence of peptides at 25 µM. Then, cells were rinsed three times with PBS, and nuclei and plasma membrane were counterstained with 1 µL/mL of Hoechst 33,342 (10 mg/mL, Thermo Fisher, Scientific, Cornellà de Llobregat, Spain) and 1 µL/mL CellMask™ deep red plasma membrane stain (5 mg/mL, Thermo Fisher Scientific, Cornellà de Llobregat, Spain), respectively. Finally, the cells were washed with PBS prior to be resuspended in PBS pH = 6.0. The experiments were performed using an Olympus Fluoview FV1000 confocal laser scanning microscope (Olympus Iberia, Hospitalet de Llobregat, Spain) equipped with Olympus Fluoview as control software. The excitation wavelengths used were 405, 488, and 658 nm to visualize the nuclei, the peptides and the plasma membrane, respectively; the wavelength of emission was 460, 510 and 690 nm, respectively. A 3D reconstruction was generated to obtain orthogonal projections using the ImageJ/fiji software.

### 4.5. Cellular Viability, Internalization, and Localization Experiments with Leishmania Parasites

The experiments were carried out with *Leishmania donovani* promastigotes (MHOM/SI/00/1S-2D strain) and *Leishmania pifanoi* amastigotes (MHOM/VE/60/Ltrod strain), respectively. Promastigotes were grown at 26 °C in RPMI medium supplemented with 10% of FCS, 2 mM l-glutamine, 20 U/mL unicilin 20 plus 48 µg/mL gentamicin.

#### 4.5.1. Cellular Viability

The viability assays were carried out using the reduction of MTT as described. The parasites were aliquoted into 96-microwell plates at a final concentration of 20 × 10^6^ cells/mL in HBSS supplemented with 10 mM d-glucose (Glc). The peptides were incubated for 4 h at the corresponding peptide concentration at 26 °C or 32 °C for promastigotes and axenic amastigotes, respectively. Afterwards, 0.5 mg/mL MTT in HBSS + 10 mM Glc was added and the cells were incubated for two additional hours. The resulting formazan was solubilized with DMSO (1% final concentration) and read at 595 nm in a Bio-Rad 640 microplate reader.

#### 4.5.2. Peptide Uptake for *Leishmania donovani* Promastigotes

The parasites were resuspended in HBSS + Glc and dispensed in 24-microwell plates (2 mL/well) at a final concentration of 20 × 10^6^ cells/mL. After incubation with the peptides for 4 h at 26 °C, the parasites were washed twice with 2 mL of HBSS + Glc plus 1% fatty-acid free bovine seroalbumin in order to remove the non-internalized peptides and resuspended in the same medium at 1 × 10^6^ cells/mL. Propidium iodide (PI) at a final concentration of 5 µg/mL was added immediately to the flow cytometric analysis to gate the viable cells exclusively. Flow cytometry was carried out in a FC500 flow cytometer, using λ_EXC_ = 488 nm and λ_EM_ = 525 nm for fluoresceinated peptides and λ_EXC_ = 488 nm.

#### 4.5.3. Intracellular Localization of the Internalized Peptides in *Leishmania donovani* Promastigotes

The parasites (2 × 10^6^ cells/mL in HBSS + Glc, 200 μL) were incubated with the corresponding peptides (2 h, 26 °C). Afterwards, they were collected from the well and washed twice with 2 mL of HBSS + Glc plus 1% bovine seroalbumin fatty-acid free. Prior to carrying out confocal microscopy, the parasites were incubated with 10 µL/mL of DAPI (4′,6-diamidine-2′-phenylindole dihydrochloride) for 10 min at 26 °C and washed twice with 2 mL of HBSS + Glc. Living parasites were observed using a Leica SP2 confocal microscope (Leica Microsistemas, S.A., Barcelona, Spain) at λ_EXC_ = 358 nm/λ_EM_ = 461 nm.

#### 4.5.4. Statistical Treatment

Statistical significance was inferred with t-test, using the statistics platform of the SigmaPlot v.11.0 software: ((*p* ≤ 0.05, (*); *p* ≤ 0.01, (**); *p* ≤ 0.001, (***)).

### 4.6. Computational Details

Geometries without steric clashes of the hybrid β, γ-peptides **8** and **11** were obtained through RDKit ETKDGv3 conformer generation [50], taking the conformer with lowest energy as the initial structure for the MD simulations. Atomic charges were computed with the restrained electrostatic potential (RESP) protocol [51]. The atom types and force field parameters were assigned through antechamber and parmchk2 tools of the AmberTools20 package [52]. The peptides were solvated with a cubic box of TIP3P water molecules and the positive charges of the guanidinium groups were neutralized with six chloride ions in each case, employing the ions94 library. The GAFF force field [53] was used for all atoms of the peptides. MD simulations were carried out under periodic boundary conditions with the OpenMM engine [54] using the OMMProtocol application [55].

The convergence of the trajectories was assessed by RMSD, all-to-all RMSD, PCA and cluster counting analyses [56], taking the exploration of the conformational space defined by the twelve backbone carbons of the peptides (see Supplementary Materials, Table S4 and Figures S1 and S2 for further details). In particular, the simulation of the β, γ-peptide **8** was extended up to 900 ns, whereas the β, γ-peptide **11** simulation was of 600 ns length. UCSF Chimera [57] was used to visually analyze the trajectories. Pytraj from the AmberTools20 package [53] was used for the hydrogen bond detection and guanidinium distance calculation. Quality threshold clustering [58], with a threshold of 2.0 Å and a selection of the alpha carbons, was used to generate the clusters and the representative structures. The SASA values of the guanidinium groups (carbon, two terminal nitrogen atoms, and their corresponding hydrogen atoms) were calculated with the MDTraj [59] implementation of the Shrake and Rupley’s algorithm.

## Figures and Tables

**Table 1:** Solvent accessible surface area (SASA) for the guanidinium groups of peptide **8** and CF-conjugate **11** along the MD simulations.

Guanidinium Group	Peptide 8		CF-Conjugate 11	
	SASA (nm^2^)	Percentage ^1^	SASA (nm^2^)	Percentage ^1^
1	1.119 ± 0.240	85.1 ± 18.3%	1.194 ± 0.186	90.8 ± 14.1%
2	1.105 ± 0.250	84.0 ± 19.0%	1.143 ± 0.194	86.9 ± 14.8%
3	1.083 ± 0.257	82.4 ± 19.5%	1.212 ± 0.164	92.2 ± 12.5%
4	1.147 ± 0.213	87.2 ± 16.2%	1.159 ± 0.201	88.1 ± 15.3%
5	1.185 ± 0.171	90.1 ± 13.0%	1.154 ± 0.196	87.8 ± 14.9%
6	1.232 ± 0.151	93.7 ± 11.5%	1.099 ± 0.229	83.6 ± 17.4%
Average	1.145 ± 0.084	87.1 ± 6.4%	1.160 ± 0.078	88.2 ± 5.9%

^1^ Percentage of SASA value of the peptide guanidinium group over a fully solvated guanidinium group (1.315 nm^2^).

## Data Availability

The data presented in this study are available on request from the corresponding author. The data are not publicly available due to their use in further studies.

## Supplementary Materials

The following are available online at https://www.mdpi.com/article/10.3390/ijms22105092/s1, Synthesis of the monomers for solid phase peptide synthesis (SPPS); NMR spectra of the new monomers; SPPS procedures; HPLC chromatograms and MS spectra of the purified peptides **6**–**11**; convergence of Molecular Dynamics simulations; Table S1. General protocol for the synthesis of the peptides. Table S2. General protocol for the derivatization of the α-amine function using the solid phase synthesis. Table S3. General protocol for the incorporation of the 5(6)-carboxyfluorescein. Table S4: Specifications of the system for the MD simulations; Figure S1: Convergence studies for the MD simulation of peptide **8**. (**a**) RMSD against the first frame of the trajectory. (**b**) Principal component analysis. Plot of the first principal component against the second principal component along the MD. (**c**) Cluster counting along the MD with a cluster cutoff of 5.0 Å. (**d**) All-to-all frames RMSD along the MD; Figure S2: Convergence studies for the MD simulation of CF-conjugate **11**. (**a**) RMSD against the first frame of the trajectory. (**b**) Principal component analysis. Plot of the first principal component against the second principal component along the MD. (**c**) Cluster counting along the MD with a cluster cutoff of 5.0 Å. (**d**) All-to-all frames RMSD along the MD.

## Abbreviations

ACN	Acetonitrile
Alloc	Allyloxycarbonyl
Amp	4-Aminoproline
CBAA	Cyclobutane Amino Acid
CF	5(6)-Carboxyfluorescein
CPP	Cell Penetrating Peptide
DAPI	4′,6-Diamidino-2-phenylindole
DCM	Dichloromethane
DDS	Drug Delivery Systems
DFT	Density Functional Theory
DIC	*N,N′*-Diisopropylcarbodiimide
DIPEA	*N*-Diisopropylethylamine
DMF	Dimethylformamide
DMSO	Dimethyl Sulfoxide
EDTA	Ethylenediaminetetraacetic Acid
ESI	Electrospray Ionization
FBS	Fetal Bovine Serum
Fmoc	Fluorenylmethyloxycarbonyl
Glc	Glucose
HBSS	Hanks Buffered Saline Solution
HPLC	High-Performance Liquid Chromatography
LPG	Lypophosphoglycan
MALDI	Matrix Assisted Laser Desorption Ionization
MS	Mass Spectroscopy
MD	Molecular Dynamics
MEM	Minimum Essential Medium
MTT	3-(4,5-Dimethylthiazol-2-yl)-2,5-diphenyltetrazolium Bromide
NMR	Nuclear Magnetic Resonance
PBS	Phosphate-Buffered Saline
PCA	Principal Component Analysis
PES	Potential Energy Surface
PI	Propidium Iodide
PyBOP	(7-Azabenzotriazol-1-yloxy)-tripyrrolidinophosphonium Hexafluorophosphate
RESP	Restrained Electrostatic Potential
RMSD	Root-Mean-Square Deviation
RMSF	Root-Mean-Square Fluctuation
RP	Reverse Phase
SASA	Solvent Accessible Surface Area
SD	Standard Deviation
SM	Supplementary Materials
SPPS	Solid Phase Peptide Synthesis
TIS	Triisopropylsilane
TOF	Time of Flight
UV	Ultraviolet Spectroscopy

## References

[1] Lindgren M., Hällbrink M., Prochiantz A., Langel Ü. (2000). Cell-penetrating Peptides. Trends Pharmacol. Sci..

[2] Langel Ü. (2002). Cell-Penetrating Peptides in Processes and Applications.

[3] Lundberg P., Langel Ü. (2003). A brief introduction to cell-penetrating peptides. J. Mol. Recognit..

[4] Kim G.C., Cheon D.H., Lee Y. (2021). Challenge to overcome current limitations of cell-penetrating peptides. Biochim. Biophys. Acta Proteins Proteom..

[5] Wu J., Li J., Wang H., Liu C.B. (2021). Mitochondrial-targeted penetrating peptide delivery for cancer therapy. Expert Opin. Drug Deliv..

[6] Vivès E., Schmidt J., Pèlegrin A. (2008). Cell-penetrating and cell-targeting peptides in drug delivery. Biochim. Biophys. Acta.

[7] Koren E., Torchillin V.P. (2012). Cell-penetrating peptides: Breaking through to the other side. Trends Mol. Med..

[8] Copolovici D.M., Langel K., Eriste E., Langel Ü. (2014). Cell-Penetrating Peptides: Design, Synthesis, and Applications. ACS Nano.

[9] Zhang D., Wang J., Xu D. (2016). Cell penetrating peptides as noninvasive transmembrane vectors for the development of new functional drug delivery systems. J. Control. Release.

[10] Dissanayake S., Denny W.A., Gamage S., Sarojini V. (2017). Recent developments in anticancer drug delivery using cell penetrating and tumor targeting peptides. J. Control. Release.

[11] Fominaya J., Bravo J., Rebollo A. (2015). Strategies to stabilize cell penetrating peptides for in vivo applications. Ther. Deliv..

[12] Berlicki Ł., Kaske M., Gutiérrez-Abad R., Bernhardt G., Illa O., Ortuño R.M., Cabrele C., Buschauer A., Reiser O. (2013). Replacement of Th^32^ and Gln^34^ in the *C*-terminal neuropeptide Y fragment 25-36 by *cis*-cyclobutane- and *cis*-cyclopentane-amino acids shifts selectivity toward the Y_4_ receptor. J. Med. Chem..

[13] Hsieh C.L., Maynard J.A., Schaub J.M., DiVenere A.M., Kuo H.C., Javanmardi K., Le K.C., Wrapp D., Lee A.G., Liu Y. (2020). Structure-based design of prefusion-stabilized SARS-CoV-2 spikes. Science.

[14] Feliciani F., Pinnen F., Stefanuccia A., Costante R., Cacciatore I., Lucente G., Mollica A. (2013). Structure-Activity Relationships of Biphalin Analogs and their Biological Evaluation on Opioid Receptors. Mini-Rev. Med. Chem..

[15] Mollica A., Costante R., Stefanucci A., Pinnen F., Lucente G., Fidanzad S., Pieretti S. (2013). Antinociceptive profile of potent opioid peptide AM94, a fluorinated analogue of biphalin with non-hydrazine linker. J. Pept. Sci..

[16] Stefanucci A., Pinnen F., Feliciani F., Cacciatore I., Lucente G., Mollica A. (2011). Conformationally Constrained Histidines in the Design of Peptidomimetics: Strategies for the χ-Space Control. Int. J. Mol. Sci..

[17] Pujals S., Giralt E. (2008). Proline-rich, amphipathic cell-penetrating peptides. Adv. Drug Deliv. Rev..

[18] Dobitz S., Aronoff M.R., Wennemers H. (2017). Oligoprolines as molecular entities for controlling distance in biological and material sciences. Acc. Chem. Res..

[19] Potocky T.B., Menon A.K., Gellman S.H. (2005). Effects of conformational stability and geometry of guanidinium display on cell entry by β-peptides. J. Am. Chem. Soc..

[20] Nagel Y.A., Raschle P.S., Wennemers H. (2017). Effect of preorganized charge-display on the cell-penetrating properties of cationic peptides. Angew. Chem. Int. Ed..

[21] Tian Y., Zeng X., Li J., Jiang Y., Zhao H., Wang D., Huang X., Li Z. (2017). Achieving enhanced cell penetration of short conformationally constrained peptides through amphiphilicity tuning. Chem. Sci..

[22] Nischan N., Herce H.D., Natale F., Bohlke N., Budisa N., Cardoso M.C., Hackenberger C.P.R. (2015). Covalent attachment of cyclic TAT peptides to GFP results in protein delivery into live cells with immediate bioavailability. Angew. Chem. Int. Ed..

[23] Lättig-Tünnemann G., Prinz M., Hoffmann D., Behlke J., Palm-Apergi C., Morano I., Herce H.D., Cardoso M.C. (2011). Backbone rigidity and static presentation of guanidinium groups increases cellular uptake of arginine-rich cell-penetrating peptides. Nat. Commun..

[24] Qian Z., Martyna A., Hard R.L., Wang J., Appiah-Kubi G., Coss C., Phelps M.A., Rossman J.S., Pei D. (2016). Discovery and mechanism of highly efficient cyclic cell-penetrating peptides. Biochemistry.

[25] Dougherty P.G., Sahni A., Pei D. (2019). Understanding Cell Penetration of Cyclic Peptides. Chem. Rev..

[26] Gutiérrez-Abad R., Carbajo D., Nolis P., Acosta-Silva C., Cobos J.A., Illa O., Royo M., Ortuño R.M. (2011). Synthesis and structural study of highly constrained hybrid cyclobutane-proline γ,γ-peptides. Amino Acids.

[27] Gorrea E., Carbajo D., Gutiérrez-Abad R., Illa O., Branchadell V., Royo M., Ortuño R.M. (2012). Searching for new cell-penetrating agents: Hybrid cyclobutane–proline γ,γ-peptides. Org. Biomol. Chem..

[28] Farrera-Sinfreu J., Giralt E., Castel S., Albericio F., Royo M. (2005). Cell-penetrating *cis*-γ-amino-L-proline-derived peptides. J. Am. Chem. Soc..

[29] Illa O., Olivares J.A., Gaztelumendi N., Martínez-Castro L., Ospina J., Abengozar M.Á., Sciortino G., Maréchal J.D., Nogués C., Royo M. (2020). Chiral cyclobutane-containing cell-penetrating peptides as selective vectors for anti-*Leishmania* drug delivery systems. Int. J. Mol. Sci..

[30] Torres E., Gorrea E., Da Silva E., Nolis P., Branchadell V., Ortuño R.M. (2009). Prevalence of eight-membered hydrogen-bonded rings in some bis(cyclobutane)-dipeptides including residues with *trans* stereochemistry. Org. Lett..

[31] Fernandes C., Faure S., Pereira E., Théry V., Declerck V., Guillot R., Aitken D.J. (2010). 12-Helix folding of cyclobutane β-amino acid oligomers. Org. Lett..

[32] Gorrea E., Pohl G., Nolis P., Celis S., Burusco K., Branchadell V., Perczel A., Ortuño R.M. (2012). Secondary structure of short β-peptides as the chiral expression of monomeric building units: A rational and predictive model. J. Org. Chem..

[33] Illa O., Olivares J.A., Nolis P., Ortuño R.M. (2017). The relevance of the relative configuration in the folding of hybrid peptides containing β-cyclobutane amino acids and γ-amino-L-proline residues. Tetrahedron.

[34] Fernandes C., Gauzy C., Yang Y., Roy O., Pereira E., Faure S., Aitken D.J. (2007). [2+2] Photocycloadditions with chiral uracyl derivatives: Access to all four stereoisomers of 2-aminocyclobutanecarboxylic acid. Synthesis.

[35] Green M., Loewenstein P.M. (1988). Autonomous functional domains of chemically synthesized human immunodeficiency virus TAT trans-activator protein. Cell.

[36] Frankel A.D., Pabo C.O. (1988). Cellular uptake of the TAT protein from human immunodeficiency virus. Cell.

[37] Vivès E., Brodin P., Lebleu B. (1997). A truncated HIV-1 TAT protein basic domain rapidly translocates through the plasma membrane and accumulates in the cell nucleus. J. Biol. Chem..

[38] Mosnann T. (1983). Rapid Colorimetric Assay for Cellular Growth and Survival: Application to Proliferation and Cytotoxicity Assays. J. Immunol. Methods.

[39] Sinclair A.N., de Graffenried C.L. (2019). More than Microtubules: The Structure and Function of the Subpellicular Array in Trypanosomatids. Trends Parasitol..

[40] Halliday C., de Castro-Neto A., Alcantara C.L., Cunha-e-Silva N.L., Vaughan S., Sunter J.D. (2021). Trypanosomatid Flagellar Pocket from Structure to Function. Trends Parasitol..

[41] Mottram J.C., Coombs G.H., Alexander J. (2004). Cysteine peptidases as virulence factors of *Leishmania*. Curr. Opin. Microbiol..

[42] Pupkis M.F., Tetley L., Coombs G.H. (1986). *Leishmania mexicana*: Amastigote hydrolases in unusual lysosomes. Exp. Parasitol..

[43] Aguilera T.A., Timmers M.M., Olson E.S., Jiang T., Tsien R.Y. (2009). Systemic in vivo distribution of activatable cell penetrating peptides is superior to cell penetrating peptides. Integr. Biol..

[44] Descoteaux A., Turco S.J. (1999). Glycoconjugates in *Leishmania* infectivity. Biochim. Biophys. Acta Mol. Basis Dis..

[45] Pae J., Liivamägi L., Lubenets D., Arukuusk P., Langel Ü., Pooga M. (2016). Glycosaminoglycans are required for translocation of amphipathic cell-penetrating peptides across membranes. Biochim. Biophys. Acta Biomembr..

[46] Nakase I., Takeuchi T., Tanaka G., Futaki S. (2008). Methodological and cellular aspects that govern the internalization mechanisms of arginine-rich cell-penetrating peptides. Adv. Drug Deliv. Rev..

[47] Vazdar M., Heyda J., Mason P.E., Tesei G., Allolio C., Lund M., Jungwirth P. (2018). Arginine “Magic”: Guanidinium Like-Charge Ion Pairing from Aqueous Salts to Cell Penetrating Peptides. Acc. Chem. Res..

[48] Homans S.W., Mehlert A., Turco S.J. (1992). Solution Structure of the Lipophosphoglycan of *Leishmania donovani*. Biochemistry.

[49] Ablan F.D.O., Spaller B.L., Abdo K.I., Almeida P.F. (2016). Charge Distribution Fine-Tunes the Translocation of a-Helical Amphipatic Peptides across Membranes. Biophys. J..

[50] Wang S., Witek J., Landrum G.A., Riniker S. (2020). Improving Conformer Generation for Small Rings and Macrocycles Based on Distance Geometry and Experimental Torsional-Angle Preferences. J. Chem. Inf. Model..

[51] Bayly C.I., Cieplak P., Cornell W.D., Kollman P.A. (1993). A well-behaved electrostatic potential based method using charge restraints for deriving atomic charges: The RESP model. J. Phys. Chem..

[52] Case D.A., Belfon K., Ben-Shalom I.Y., Brozell S.R., Cerutti D.S., Cheatham T.E., Cruzeiro V.W.D., Darden T.A., Duke R.E., Giambasu G. (2020). Amber 2020.

[53] Wang J., Wolf R.M., Caldwell J.W., Kollman P.A., Case D.A. (2004). Development and testing of a general Amber force field. J. Comput. Chem..

[54] Eastman P., Swails J., Chodera J.D., McGibbon R.T., Zhao Y., Beauchamp K.A., Wang L.P., Simmonett A.C., Harrigan M.P., Stern C.D. (2017). OpenMM 7: Rapid development of high-performance algorithms for molecular dynamics. PLoS Comput. Biol..

[55] Rodríguez-Guerra Pedregal J., Alonso-Cotchico L., Velasco-Carneros L., Maréchal J.-D. (2018). OMMProtocol: A command line application to launch molecular dynamics simulations with OpenMM. ChemRxiv.

[56] Sciortino G., Sánchez-Aparicio J.-E., Rodríguez-Guerra Pedregal J., Garribba E., Maréchal J.-D. (2019). Computational insight into the interaction of oxaliplatin with insulin. Metallomics.

[57] Pettersen E.F., Goddard T.D., Huang C.C., Couch G.S., Greenblatt D.M., Meng E.C., Ferrin T.E. (2004). UCSF Chimera—A visualization system for exploratory research and analysis. J. Comput. Chem..

[58] González-Alemán R., Hernández-Castillo D., Caballero J., Montero-Cabrera L.A. (2020). Quality Threshold Clustering of Molecular Dynamics: A Word of Caution. J. Chem. Inf. Model..

[59] McGibbon R.T., Beauchamp K.A., Harrigan M.P., Klein C., Swails J.M., Hernández C.X., Schwantes C.R., Wang L.-P., Lane T.J., Pande V.S. (2015). MDTraj: A Modern Open Library for the Analysis of Molecular Dynamics Trajectories. Biophys. J..

