# Supplementary material for: Hybrid Cyclobutane/Proline-Containing Peptidomimetics: The Conformational Constraint Influences Their Cell-Penetration Ability
Source: Int J Mol Sci. 2021 May 11;22(10):5092. doi: 10.3390/ijms22105092 (PMC8151717; doi:10.3390/ijms22105092)
Supplement: Supplementary file 1 [file ijms-22-05092-s001.zip › ijms-1170174-supplementary.pdf]

SUPPLEMENTARY MATERIALS

Hybrid Cyclobutane/Proline-Containing Peptidomimetics: The Conformational Constraint Influences Their Cell-Penetrating Ability

Ona Illa, Jimena Ospina, José-Emilio Sánchez-Aparicio, Ximena Pulido, María Ángeles Abengozar, Nerea Gaztelumendi, Daniel Carbajo, Carme Nogués, Luis Rivas, Jean-Didier Maréchal, Miriam Royo and Rosa M. Ortuño

Table of contents

Synthesis of the monomers for solid phase peptide synthesis (SPPS) 2
NMR spectra of the new monomers 4
SPPS procedures 7
HPLC chromatograms and MS spectra of the purified peptides 10
Convergence of molecular dynamics simulations 18
References 20

Synthesis of the Monomers for SPPS

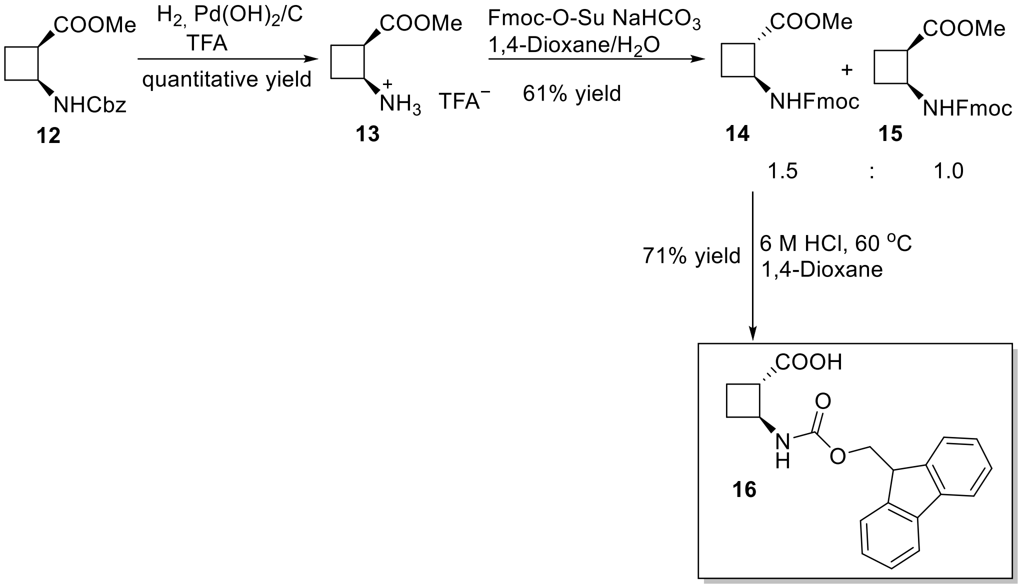

Synthesis of (1*S*,2*S*)- and (1*R*,2*S*)-2-(9H-fluoren-9-ylmethyloxycarbonylamino)-cyclobutane-1-carboxylic acid methyl ester (14) and (15) respectively

Compound **12** [1] (345 mg, 1.31 mmol) was dissolved in the minimum amount of MeOH and hydrogenated under 7 atm of pressure in the presence of $Pd(OH)_2/C$ (34 mg) and TFA (0.1 mL, 1.31 mmol) overnight. Then, the crude was filtered through Celite and the solvent was evaporated. The resulting ammonium salt **13** (mg, 1.31 mmol) was used in the following step without further purification. Amine **13** (300 mg, 1.31 mmol) was dissolved in a 1:1 water-dioxane mixture (20 mL) at 0 °C. Then, $NaHCO_3$ (0.33 g, 3.94 mmol) was added, keeping the pH value between 8 and 9. Next, Fmoc-OSu (0.44 g, 1.31 mmol) was added. The resulting mixture was stirred at 0 °C for 4 h. Then the solvent was evaporated. The crude was diluted in EtOAc (50 mL) and washed with 0.1 M HCl (30 mL) and brine (30 mL). The organic layer was dried with $MgSO_4$ and the solvent was evaporated under vacuum. The crude was purified by column chromatography on silica gel using 2:1 hexane-EtOAc as eluent. Compounds **14** (165 mg, 0.47 mmol, 36% yield) and **15** (115 mg, 0.31 mmol, 25% yield) were obtained as white solids.

Compound 14: $[\alpha]_D$: + 5.1 (c = 1.1, MeOH); mp.: 152–154 °C (MeOH); IR (ATR): ν 3338, 2960, 2953, 1685, 1535 cm^{-1}; ^1H NMR (250 MHz, $CDCl_3$) δ 1.85–2.09 (m, 3H, H_{4a}, H_{3a}, H_{3b}), 2.18–2.35 (m, 1H, H_{4b}), 3.02–3.19 (m, 1H, H_1), 3.71 (s, 3H, CH_3), 4.14–4.36 (m, 2H, H_2, CH_{Fmoc}), 4.37–4.50 (m, 2H, CH_{2Fmoc}), 4.97–5.13 (m, 1H, NH), 7.30–7.83 (m, 8H, $H_{Ar\ Fmoc}$); ^{13}C NMR (90 MHz, $CDCl_3$) δ 17.5 (C_4), 30.6 (C_3), 45.5 (C_1), 46.2 (CH_{Fmoc}), 47.57 (OCH_3), 51.0 (C_2), 51.5 (CH_{2Fmoc}), 128.2, 131.2 ($8C_{Ar}$), 141.6, 144.3 (4C, C_{Fmoc}), 155.7 ($CO_{carbamate}$), 177.6 ($CO_{carbamate}$), 177.6 (CO_2CH_3). HRMS: Calculated for $C_{27}H_{25}NNaO_4$ [M + Na]$^+$: 374.1363; Experimental: 374.1358. **Compound 15**: $[\alpha]_D$: −25.1 (c = 1.3, MeOH); mp.: 85–87 °C (MeOH); IR (ATR): ν 3338, 2953, 2960, 1692, 1535 cm^{-1}; ^1H NMR (250 MHz, $CDCl_3$) δ 1.96–2.11 (m, 2H, H_{4a}, H_{3a}), 2.27–2.49 (m, 2H, H_{3b}, H_{4b}), 3.36–3.50 (m, 1H, H_1), 3.73 (s, 3H, CH_3), 4.14–4.29 (m, 1H, H_2), 4.32–4.47 (m, 2H, CH_{2Fmoc}), 4.52–4.68 (m, 1H, CH_{Fmoc}), 5.66–5.82 (m, 1H, NH), 7.31–7.85 (m, 8H, H_{Ar} Fmoc) ppm; ^{13}C NMR (90 MHz, $CDCl_3$) δ 19.1 (C_4), 30.1 (C_3), 45.5 (C_1), 46.5 (CH_{Fmoc}), 47.6 (CH_3), 52.2 (C_2), 67.3, 67.9 (CH_{2Fmoc}), 120.3, 125.4, 127.4, 128.0 ($8C_{Ar}$), 141.6, 144.3 (4C, C_{Fmoc}), 155.7 ($CO_{carbamate}$), 172.2 (CO_2CH_3) ppm; HRMS: Calculated for $C_{27}H_{25}NNaO_4$ [M+Na]$^+$: 374.1363; Experimental: 374.1357.

Synthesis of (1*S*,2*S*)-2-(9H-fluoren-9-ylmethyloxycarbonylamino)-cyclobutane-1-carboxylic acid (16)

Amino acid **14** (0.22 g, 0.63 mmol) was dissolved in dioxane (13 mL). Then 6 M HCl (13 mL, 78 mmol) was added. The mixture was heated at 60 °C for 3 h. Then the solvent was evaporated under vacuum. The crude was purified by column chromatography on silica gel using 2:1:0.1 hexane-EtOAc-CH_3CO_2H as eluent. Compound **16** (0.15 g, 0.44 mmol, 71% yield) was obtained as a white solid. $[\alpha]_D$: + 11.9 (*c* = 0.9, MeOH); m.p.: 113-115 °C (MeOH); IR (ATR): ν 3329, 2953, 1685, 1535, 1535, 1261 cm^{-1}; ^1H NMR (250 MHz, $CDCl_3$) δ 1.74–1.98 (m, 1H, H_{4a}), 2.07–2.39 (m, 3H, $H_{3a,}$ $H_{4b,}$ H_{4b}), 2.98–3.22 (m, 1H, H_1), 4.06-4.31 (m, 2H, H_2, $H_{2'}$), 4.50–4.54 (d,^3J = 6 Hz, 2H, $H_{1'}$), 5.19 (broad s, 1H, NH), 7.40 (dt, ^2J = 24, $^3J'$= 7 Hz, 4H, $H_{5',6'Ar\ Fmoc}$), 7.60 (d, ^2J = 7 Hz, 2H, $H_{4'Ar\ Fmoc}$), 7.80 (d, ^2J = 7 Hz, 2H, $H_{7'Ar\ Fmoc}$) ppm; ^{13}C NMR (90 MHz, $CDCl_3$) δ 18.6 (C_4), 24.7 (C_3), 46.8 (C_1), 48.9 ($C_{2'}$), 52.2 (C_2), 67.2 ($C_{1'}$), 119.8, 120.1 ($C_{4'}$), 124.7 (2C, $C_{5'}$), 126.9, 127.1 ($C_{7'}$), 127.6, 128.2 ($C_{6'}$), 141.1 (2C, $C_{8'}$), 143.2 (2C, C*3′*), 156.7 ($CO_{carbamate}$), 174.8 (CO_{acid}) ppm; HRMS: Calculated for $C_{20}H_{19}NNaO_4$ $[M + Na]^+$: 360.1206; Experimental: 360.1207.

(2*S*,4*R*)-4-(9H-fluoren-9-ylmethyloxycarbonylamino)-1-(allyloxycarbonyl)-pyrrolidine-2-carboxylic acid (18)

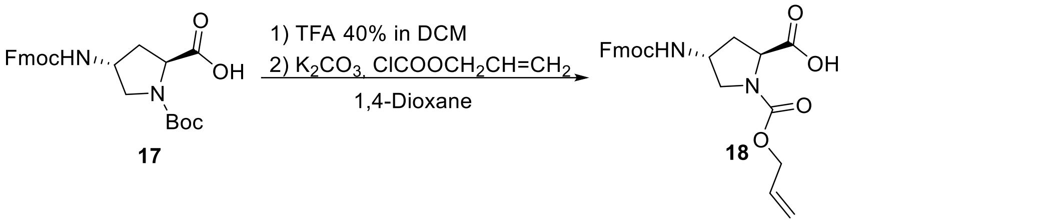

Commercially available *trans*-γ-amino-L-proline **17** (2.00 g, 4.42 mmol) was dissolved in a 40% solution of TFA in dichloromethane (25 mL). The resulting mixture was stirred at room temperature for 30 minutes. The solvent and excess volatiles were evaporated under vacuum (coevaporations with 2 × 10 mL of DCM and 1 × 10 mL Et_2O). The intermediate ammonium salt (2.06 g, 4.42 mmol, quantitative yield) was obtained as a white solid and used without further purification. Then, the ammonium salt (2.06 g, 4.42 mmol) was dissolved in dioxane (20 mL) and 25% K_2CO_3 aqueous solution (48 mL) and allyl chloroformate (0.56 mL, 5.30 mmol) were added. The reaction mixture was stirred at room temperature for 2 hours, acidified with 2 M HCl and extracted with EtOAc (3 × 30 mL). The organic layers were combined, dried over $MgSO_4$, and the solvent was evaporated under vacuum to afford compound **18** (1.73 g, 3.96 mmol, 90% yield) whose physical constants and spectroscopic data agree with those previously described [2].

NMR SPECTRA of the New MONOMERS

Compound 14

^{1}H-NMR (250 MHz, $CDCl_3$)

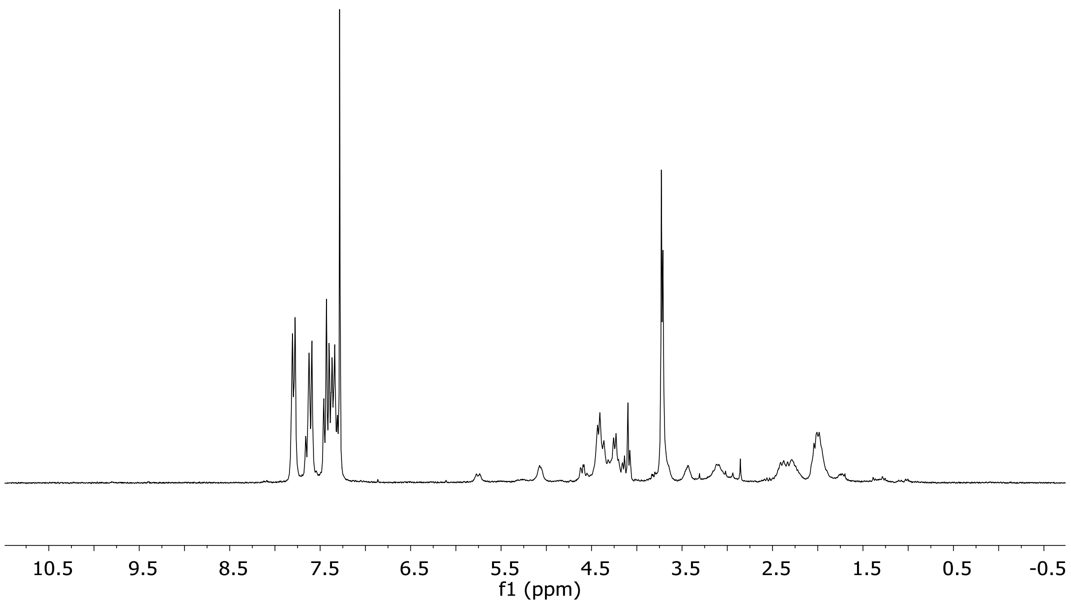

^{13}C-NMR (90 MHz, $CDCl_3$)

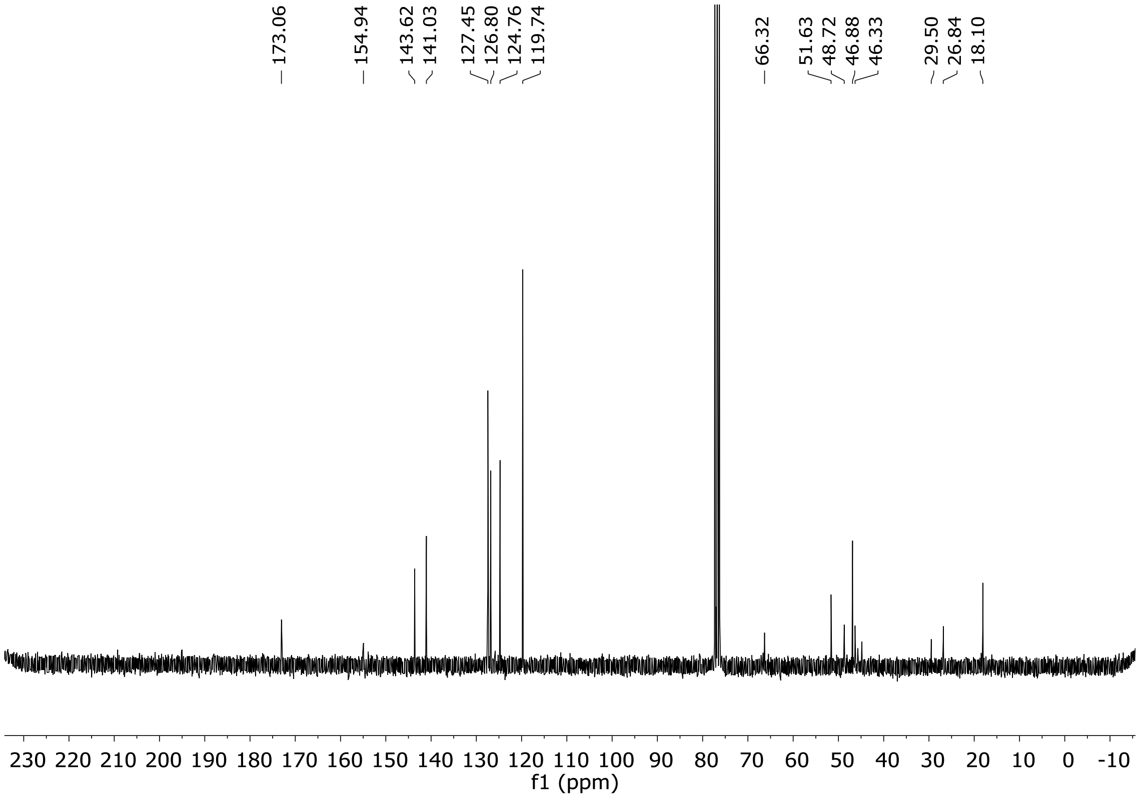

Compound 15
^{1}H-NMR (250 MHz, CDCl$_3$)

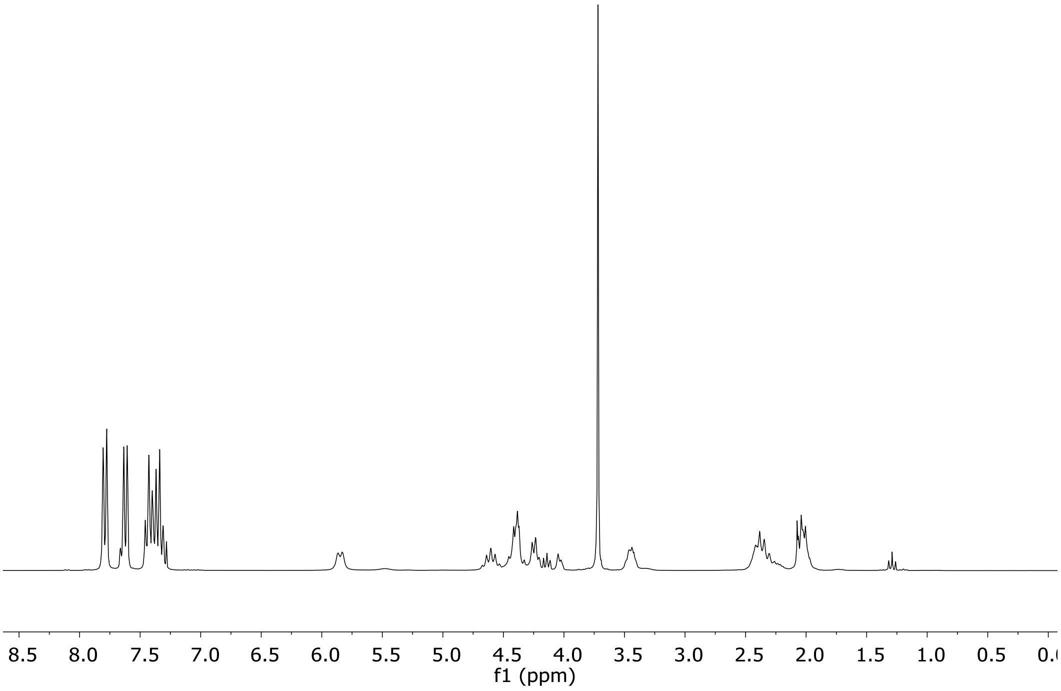

^{13}C-NMR (90 MHz, CDCl$_3$)

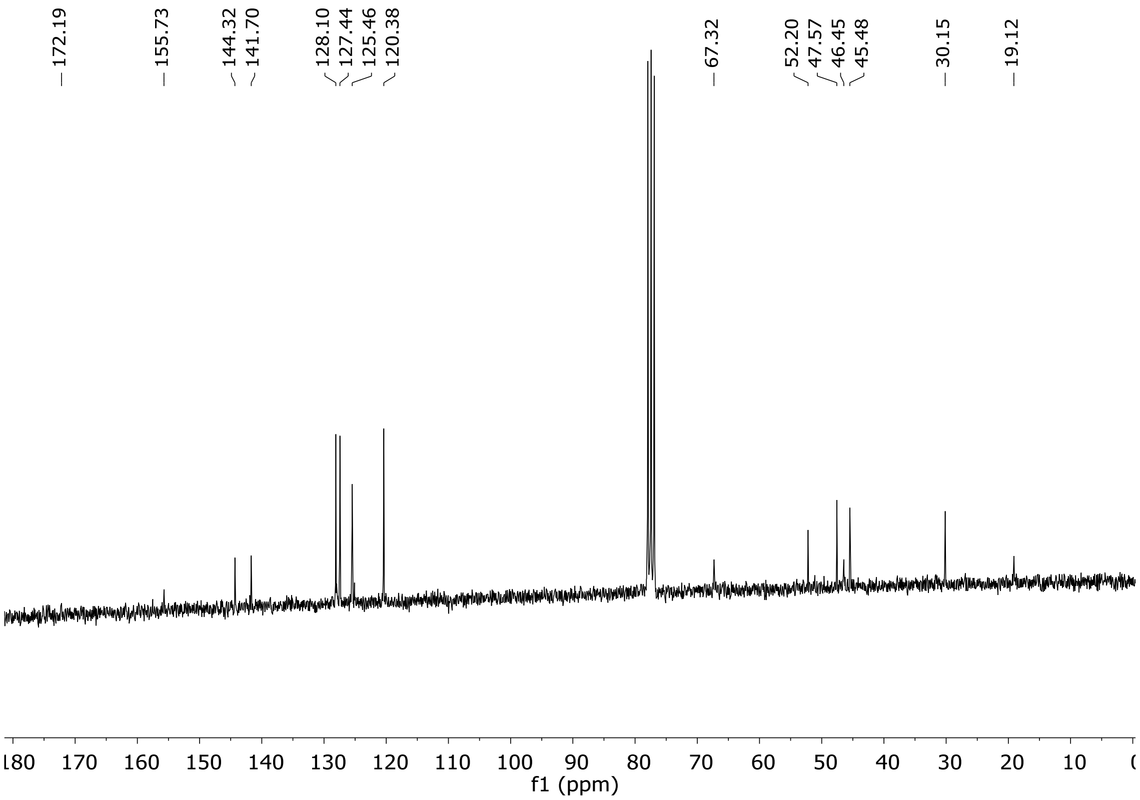

Compound 16
^{1}H-NMR (250 MHz, $CDCl_3$)

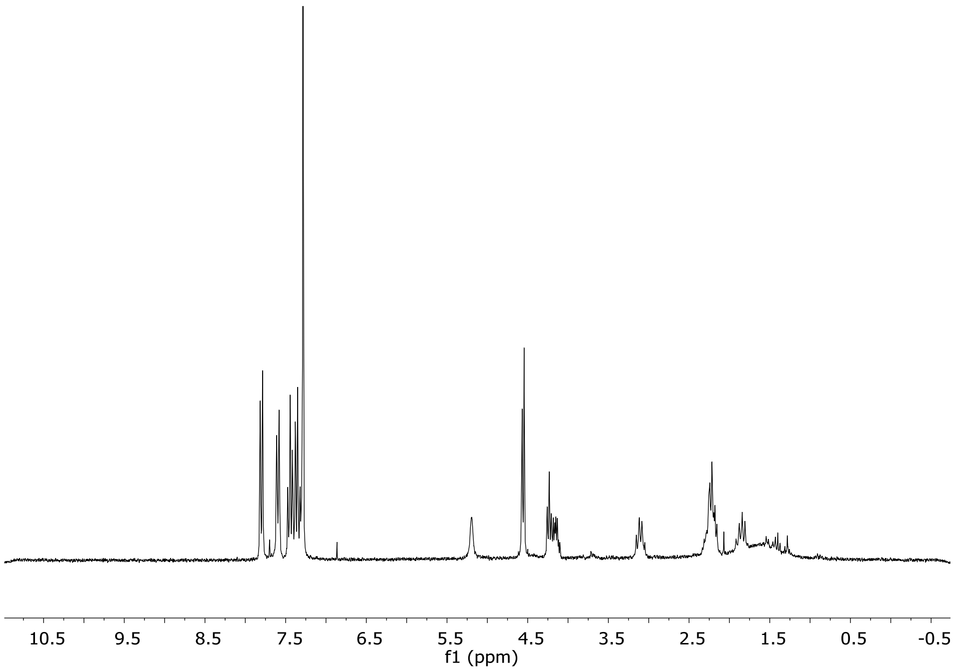

SPPS PROCEDURES

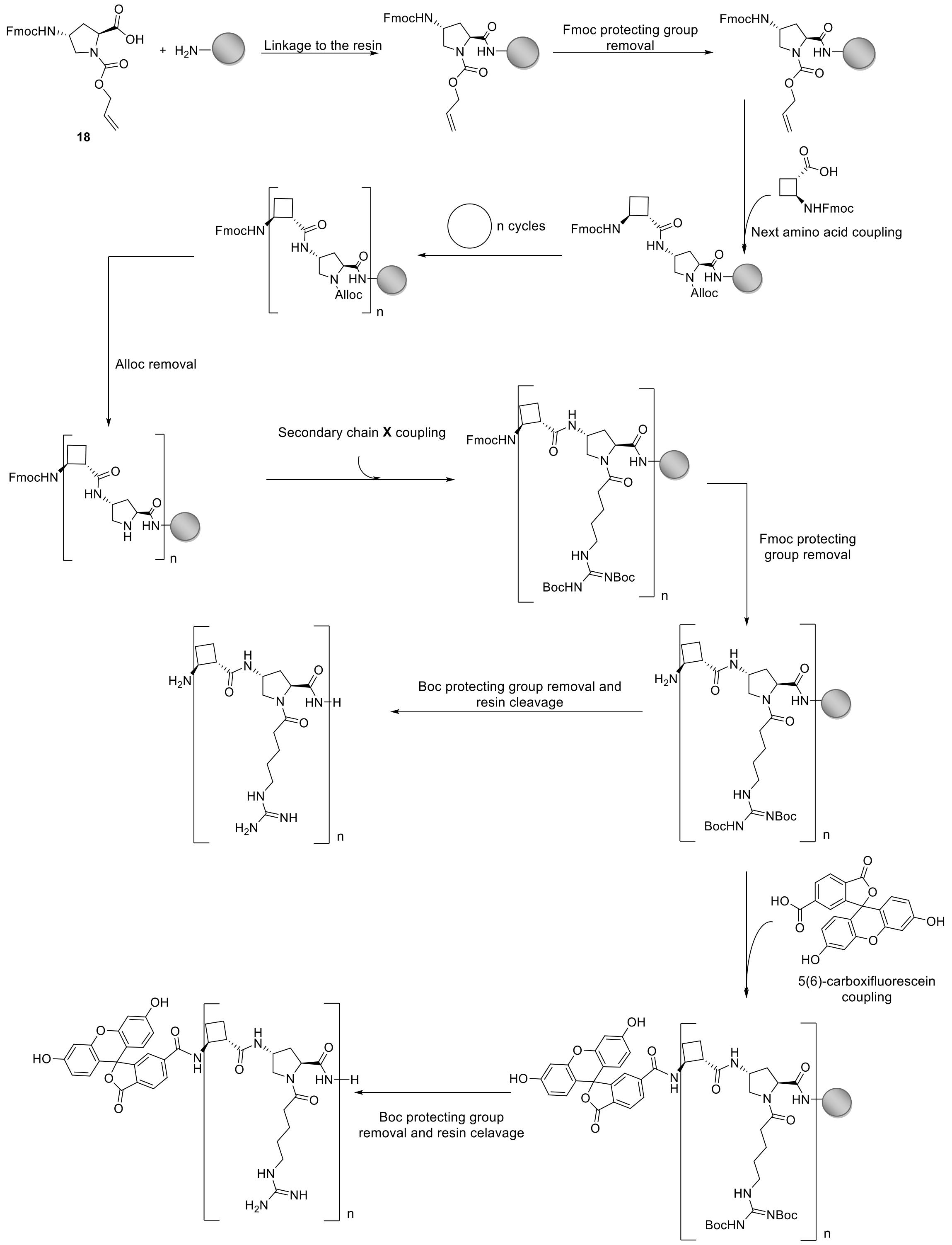

Scheme 1. Protocol for the SPPS of the β,γ-peptides **6–8** and the CF-β,γ-peptides **9–11**.

Aminomethyl-ChemMatrix® resin with 0.74 mmol/g functionalization was used. It was conditioned with successive washes with DCM, TFA / DCM (4:5, *v/v*), DIPEA / DCM (4:5, *v/v*), DMF, and DCM. The presence of primary amines was verified and then the linker was coupled using 3:3:3 Fmoc-Rink amide-Linker/ DIC / OxymaPure® in DMF for 1 h. The Fmoc protecting group was removed with 20% piperidine in DMF (3 × 10 min). The Fmoc / Alloc strategy was used. The protocol is summarized in Supplementary Table S1.

Supplementary Table S1. General protocol for the synthesis of the peptides.

Step	Reagents/Solvents	Aim	Cycles	t/Cycle (min.)
1	DCM	Wash	5	1
2	DMF	Wash	5	1
3	(2*S*,4*R*)-Fmoc-amino-proline/DIC/HOBt (3:3:3) in DMF	Coupling	1	120
4	DMF	Wash	5	1
5	DCM	Wash	5	1
6	Ninhydrine test (−)	Coupling test	1	3
7	Piperidine/DMF (2:8, *v*/*v*)	Deprotection	3	10
8	DMF	Wash	5	1
9	DCM	Wash	5	1
10	Ninhydrine test (+)	Deprotection test	1	3
11	DMF	Wash	5	1
12	(1*S*,2*S*)-Fmoc-amino-cycloBu/HOBt/ PyBOP/DI-PEA (3:3:3:6) in DMF	Coupling	1	120
13	DMF	Wash	5	1
14	DCM	Wash	5	1
15	Ninhydrine test (−)	Coupling test	1	3
16	Piperidine/DMF (2:8, *v*/*v*)	Wash	3	10
17	DMF	Wash	5	1
18	DCM	Wash	5	1
19	Ninhydrine test (+)	Deprotection test	1	3

Steps from 1 to 19 were repeated n (n = 4, 5, and 6) times to obtain the octamer, decamer. and dodecamer peptides, respectively. By the time the desired peptide was obtained, 200 mg of resin were separated for further reactions.

Once the peptide skeleton was prepared, the derivatization of the α-amino function was performed. In a first step, the Alloc protecting groups were removed by catalytic reduction using palladium, and then the guanidinylated side-chain (5-(2,3-bis(*tert*-butoxycarbonyl)guanidino)pentanoic acid), previously synthesized in solution [3], was incorporated using DIC / OxymaPure® as coupling agents. After that, the Fmoc group of the terminal residue was removed. The protocol is summarized in Supplementary Table S2.

Supplementary Table S2. General protocol for the derivatization of the α-amine function using the solid phase synthesis.

Step	Reagents/Solvents	Aim	Cycles	t/Cycle (min.)
1	DCM	Wash	5	1
2	$PhSiH_3/Pd(PPh_3)_4$ (12:0.1) in DCM	Deprotection	2	15
5	DCM	Wash	5	1
6	DMF	Wash	5	1
7	$(Et)_2NCSSNa\ 3H_2O$ (20 mM in DMF)	Palladium wash	5	1
8	DMF	Wash	5	1
9	5-(2,3-bis(*tert*-butoxycarbonyl)guanidino)pentanoic acid/DIC/OxymaPure® (5:5:5) for each proline	Coupling	1	120
10	DMF	Wash	5	1
11	DCM	Wash	5	1
12	DMF	Wash	5	1
13	Piperidine/DMF (2:8, *v*/*v*)	Deprotection	3	10
14	DMF	Wash	5	1

15	DCM	Wash	5	1
16	DMF	Wash	5	1

Once the desired length peptides were prepared and functionalized, the resin was divided into two parts, of which 100 mg were left as amino free and the other 100 mg were coupled to carboxyfluorescein (CF) through the terminal amino group (see the summarized Protocol in Supplementary Table S3).

Supplementary Table S3. General protocol for the incorporation of the 5(6)-carboxyfluorescein.

Step	Reagents/Solvents	Aim	Cycles	t/Cycle (min.)
1	DMF	Wash	5	1
2	CF/ OxymaPure®/PyBOP/DIPEA (4:6:4:6) in DMF	Coupling	1	120
3	DMF	Wash	5	1
4	DCM	Wash	5	1
5	Ninhydrine test (−)	Deprotection test	1	3
6	TFA/(^{i}Pr)$_3$SiH/H_2O (95:2.5:2.5)	Deprotection/Resin cleavage	1	120
7	DCM	Wash	5	1

Cleavage from the Aminomethyl-ChemMatrix® Resin and Removal of the Boc Carbamate Protecting Groups: Acid Hydrolysis

The cleavage of the peptide from the resin was carried out through acid hydrolysis using TFA/TIS/H_2O (95:2.5:2.5) during 3 h under stirring. The peptide was separated from the resin through filtration. The solid was washed with DCM (4×). The solution was concentrated under vacuum but not until dryness. Then, the peptide was precipitated through addition of cold Et_2O. The solid was filtered and centrifuged with Et_2O (3×). The resulting solid was dissolved in CH_3CN/H_2O (1:1, *v*/*v*) and lyophilized.

Cleavage from the Aminomethyl-ChemMatrix® Resin: Acid Hydrolysis

The cleavage of the peptide from the resin was carried out through acid hydrolysis using TFA/TIS/H_2O (95:2.5:2.5) and stirring for 3 h. The peptide was separated from the resin through filtration. The solid was washed with DCM (4×). The solution was concentrated under vacuum but not until dryness. Then, the peptide was precipitated through addition of cold Et_2O. The solid was filtered and centrifuged with Et_2O (3×). The resulting solid was dissolved in CH_3CN/H_2O (1:1, *v*/*v*) and lyophilized.

Cleavage from the H-Rink Amide ChemMatrix®Resin

The same conditions as for the Aminomethyl-ChemMatrix® resin were used.

HPLC CHROMATOGRAMS AND MS SPECTRA OF PURIFIED PEPTIDES

Peptide 6:

RP-HPLC

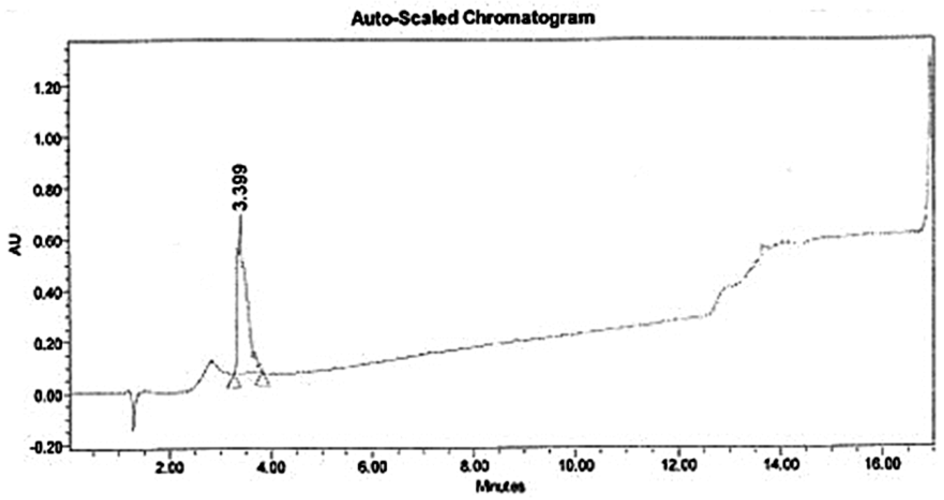

Peak Results

	Name	RT	Area	Height	Amount	Units
1		3.399	6971712	556141		

MALDI-TOF

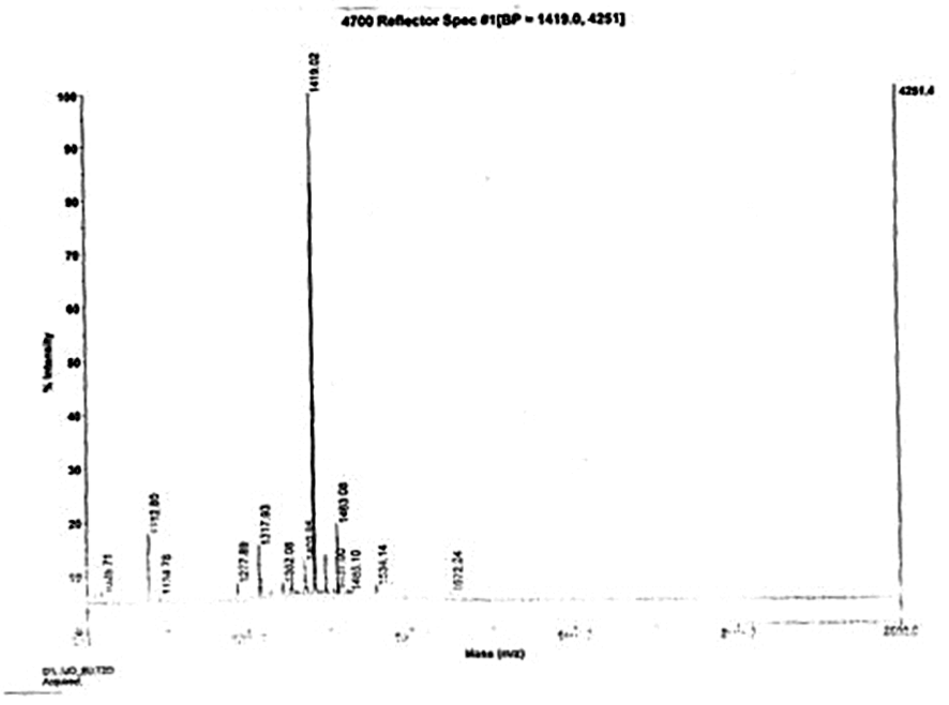

<u>Peptide 7:</u>

RP-HPLC:

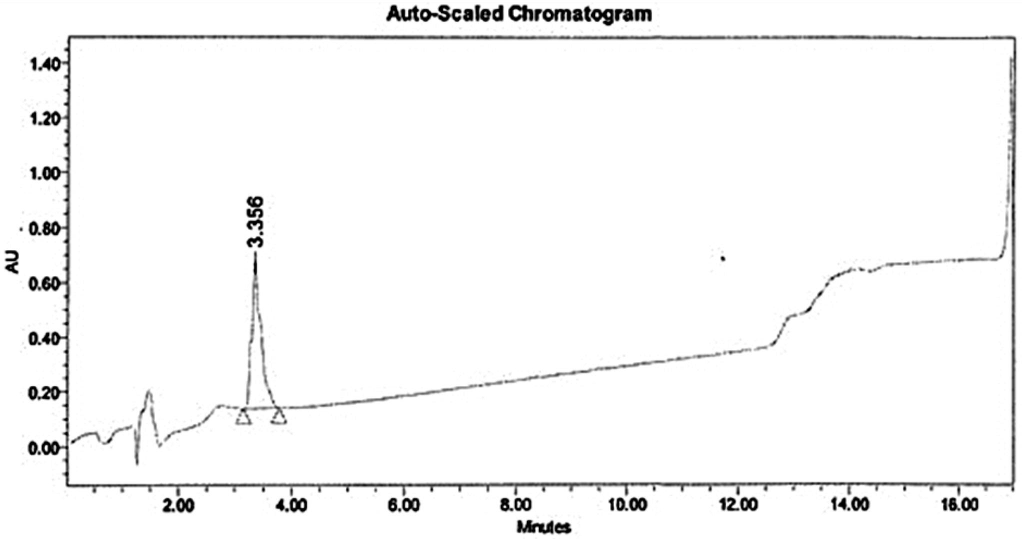

Peak Results

	Name	RT	Area	Height	Amount	Units
1		3.356	6050804	536898		

MALDI-TOF:

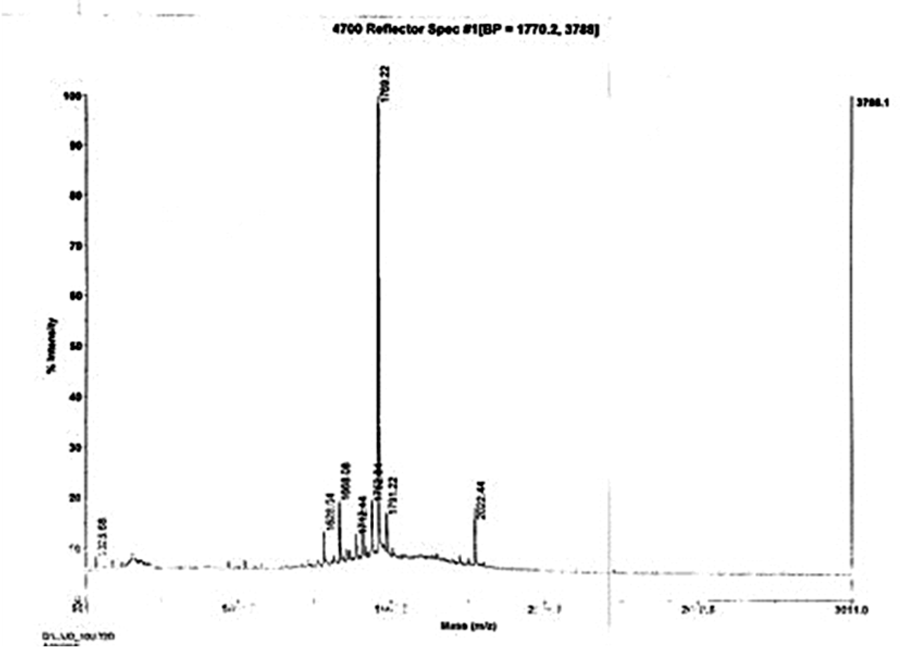

Peptide 8:

RP-HPLC:

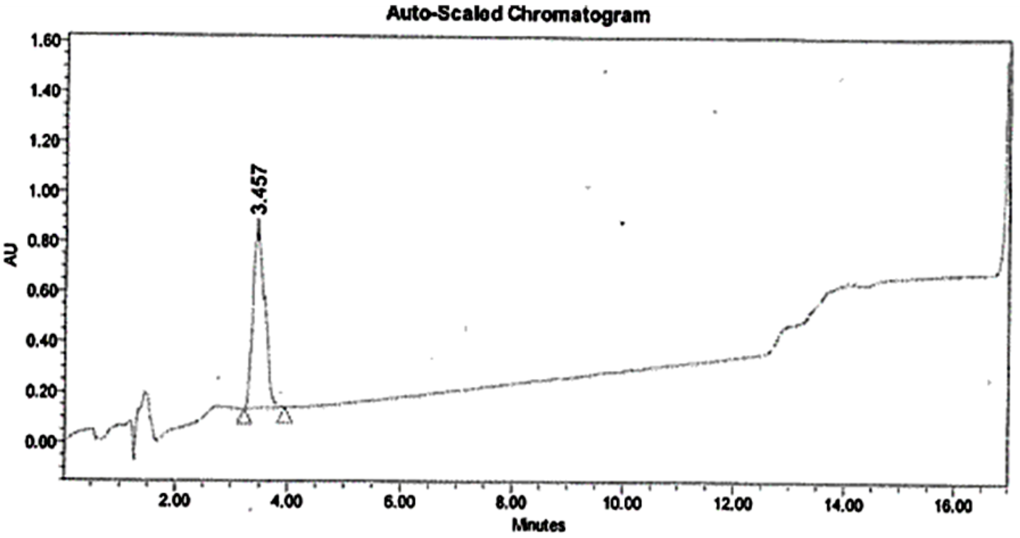

Peak Results

	Name	RT	Area	Height	Amount	Units
1		3.457	10507154	715084		

MALDI-TOF:

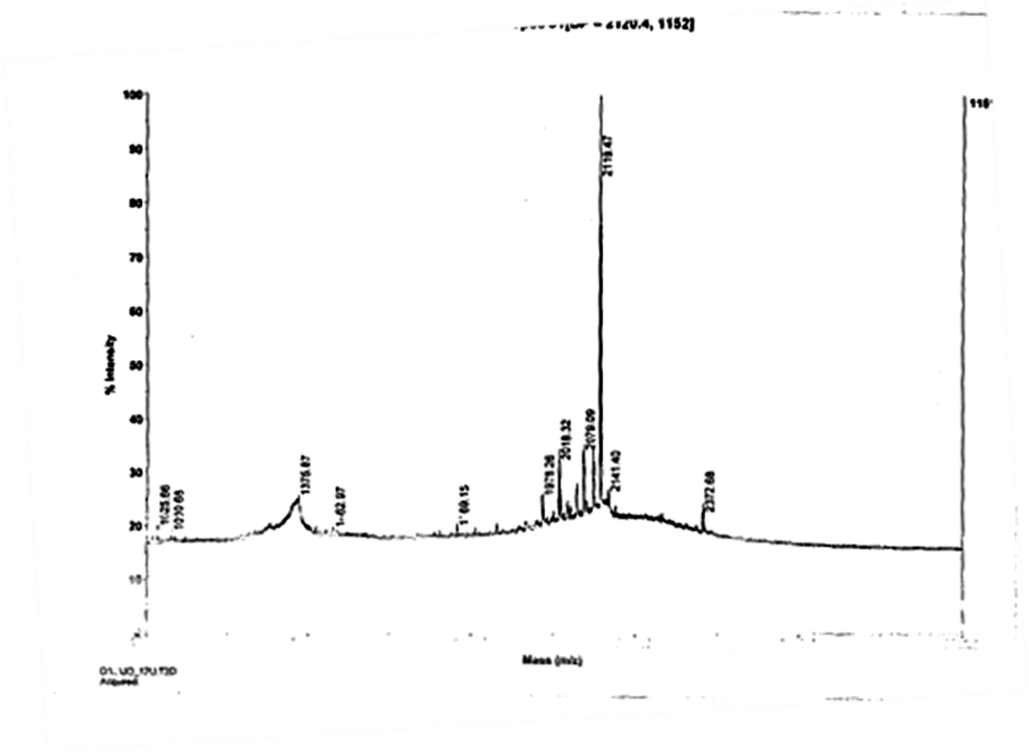

Peptide 9:

RP-HPLC:

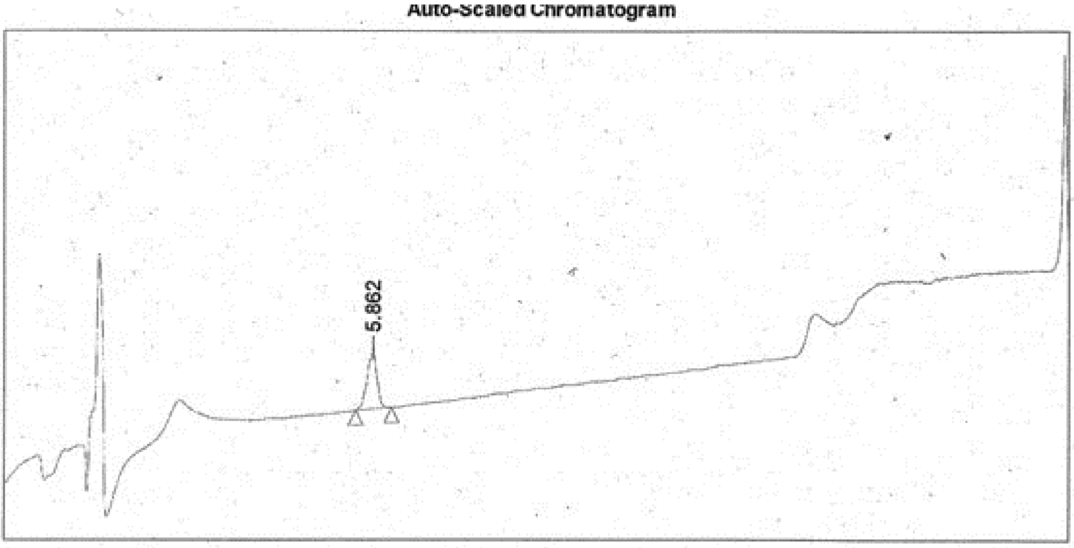

Peak Results

	Name	RT	Area	Height	Amount	Units
1		5.862	1858915	180325		

m/*z* **(ESI):**

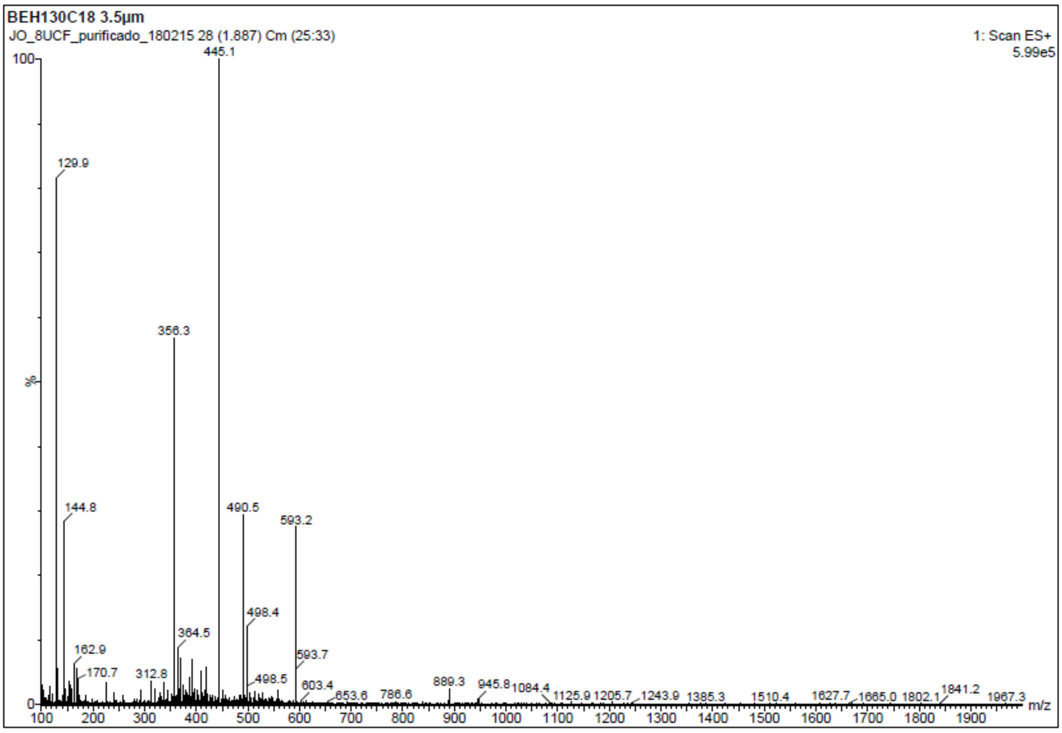

MALDI-TOF:

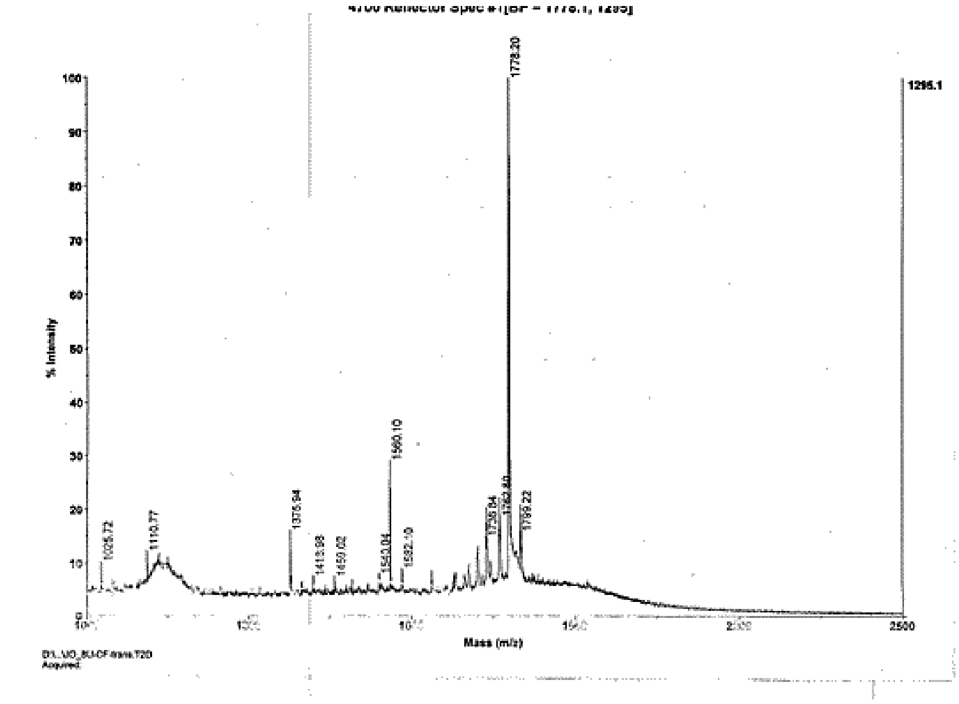

Peptide 10:

RP-HPLC:

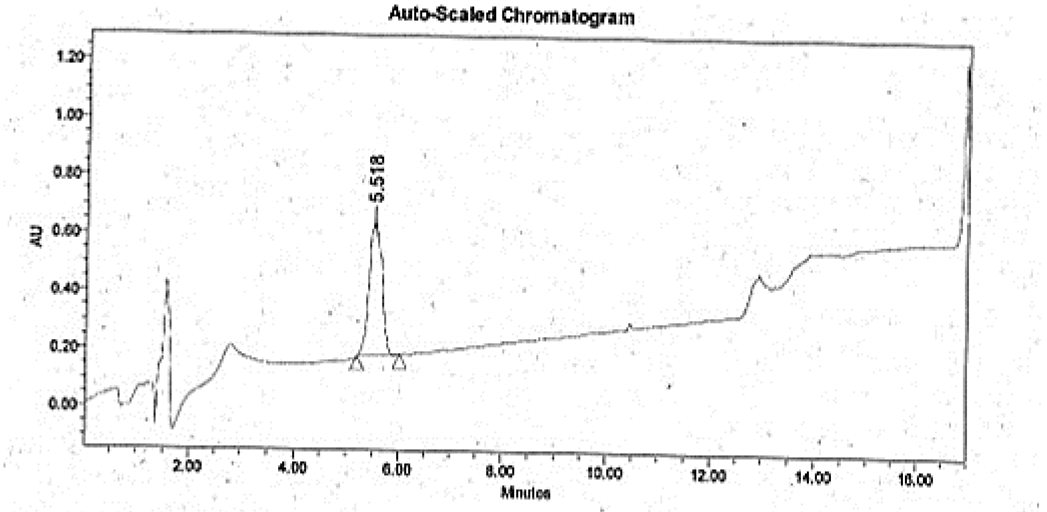

Peak Results

	Name	RT	Area	Height	Amount	Units
1		5.518	7757108	470675		

m/z (ESI):

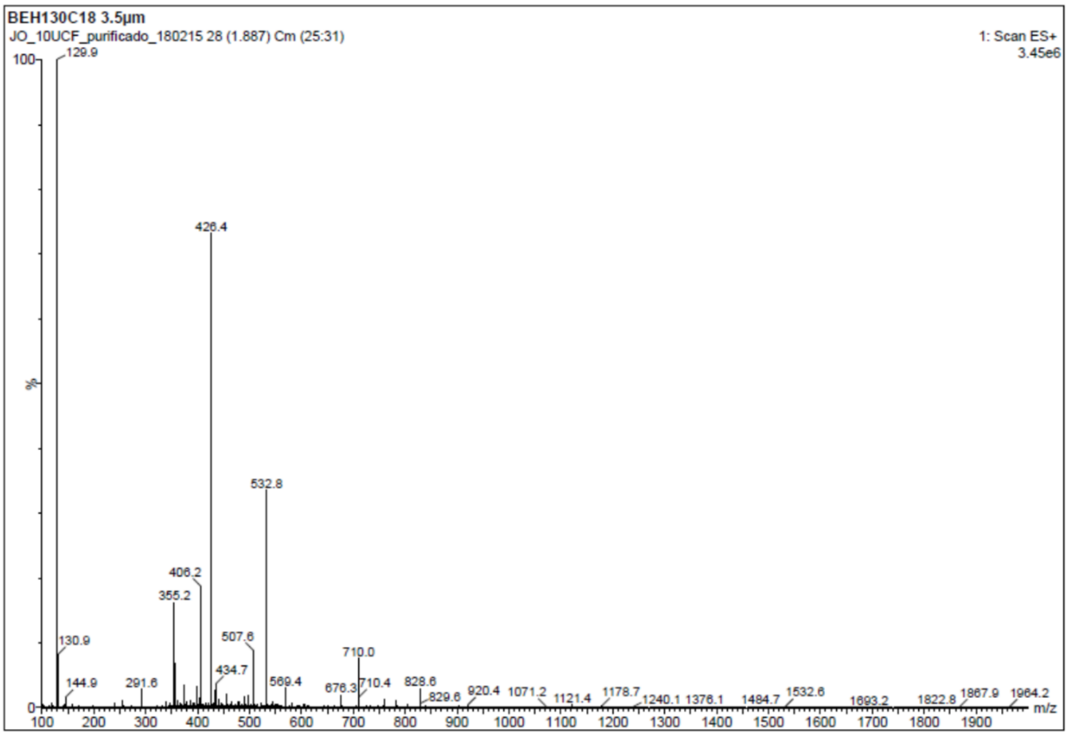

MALDI-TOF:

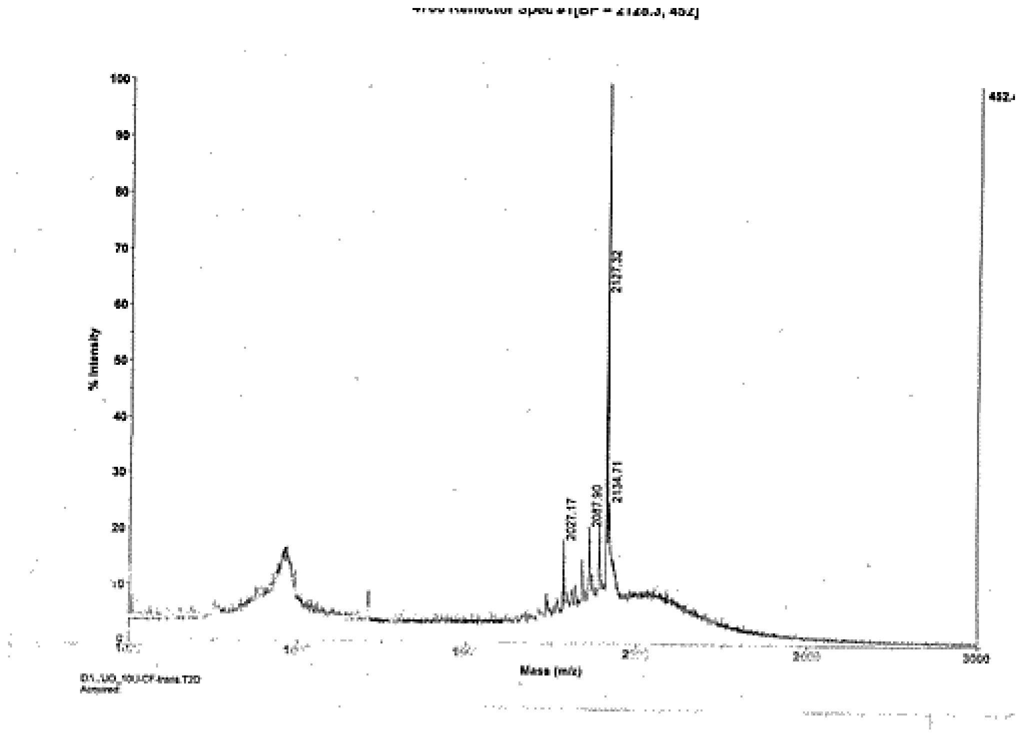

Peptide 11:

RP-HPLC:

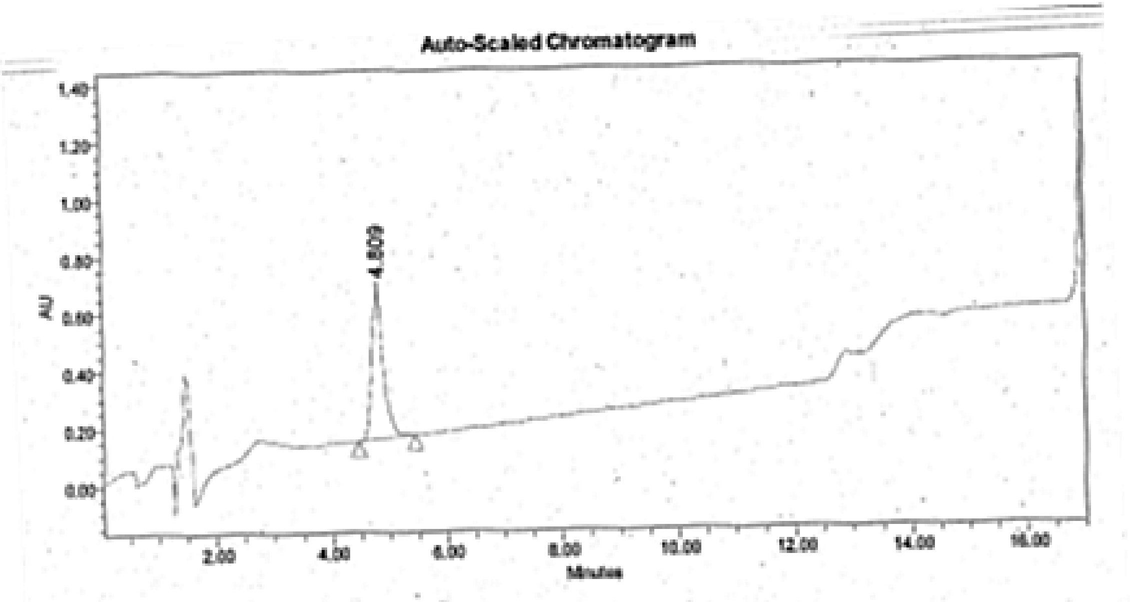

Peak Results

	Name	RT	Area	Height	Amount	Units
1		4.809	6951035	512210		

m/z **(ESI):**

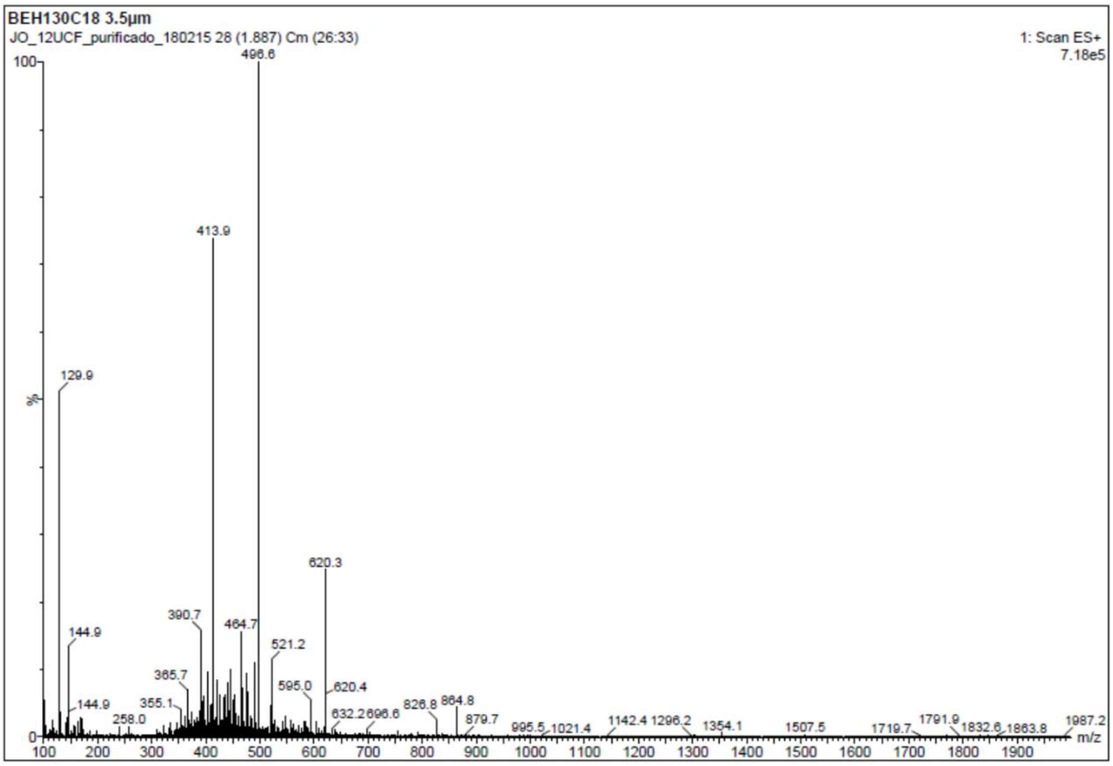

MALDI-TOF:

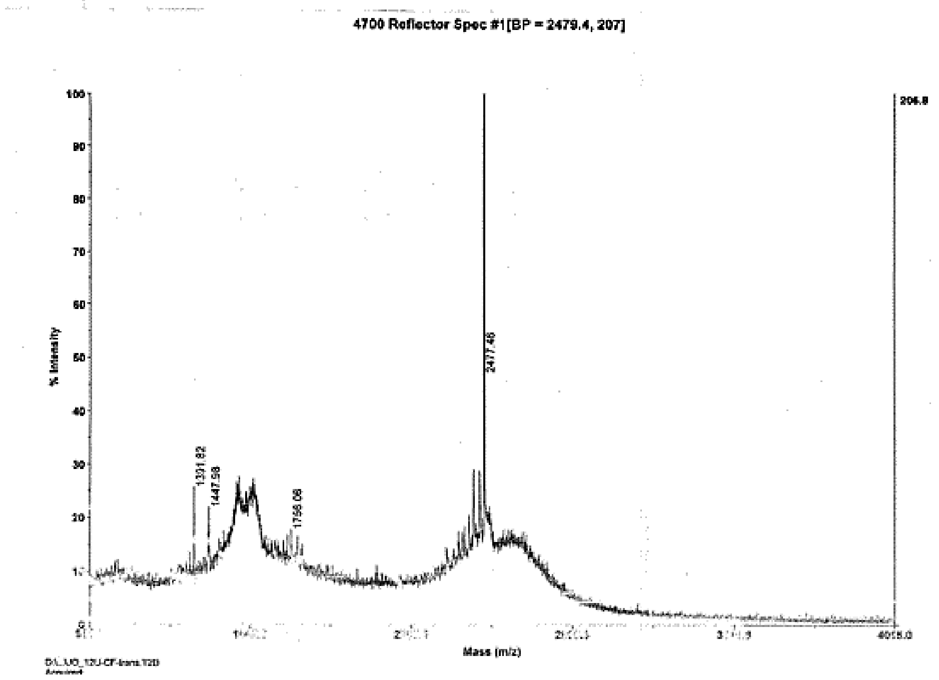

4700 Reflector Spec #1[BP = 2479.4, 207]
206.8
% Intensity
100
90
80
70
60
50
40
30
20
1331.82
1447.58
1756.08
2477.46
4005.0
Mass (m/z)

CONVERGENCE OF MOLECULAR DYNAMICS SIMULATIONS

For peptide **8** and its CF-conjugate **11**, the production trajectory of the MD simulations was preceded by the following stages: i) 1000 energy minimization steps holding the peptide fixed in order to relax the water box; ii) 2500 energy minimization steps on the whole system; iii) thermalization of the water molecules by increasing the temperature from 0 K to 300 K, while keeping the peptide fixed; and iv) thermalization of the peptide at 300 K. A summary of the specifications for each MD is provided in Supplementary Table S4.

Supplementary Table S4. Specifications of the system for the MD simulations.

Peptide	Simulation Time (ns)	Number of Atoms (peptide)	Water Molecules	Counterions (Cl$^-$)
β,γ **8**	900	316	4686	6
CF-β,γ **11**	600	353	4769	6

To determine the length of each simulation, the recurrence in the exploration of the same conformational space for a statistically relevant number of times was considered an indicator of enough sampling. Several convergence studies were carried out on each simulation (Supplementary Figures S1,S2), always considering the backbone carbon atoms of the peptide.

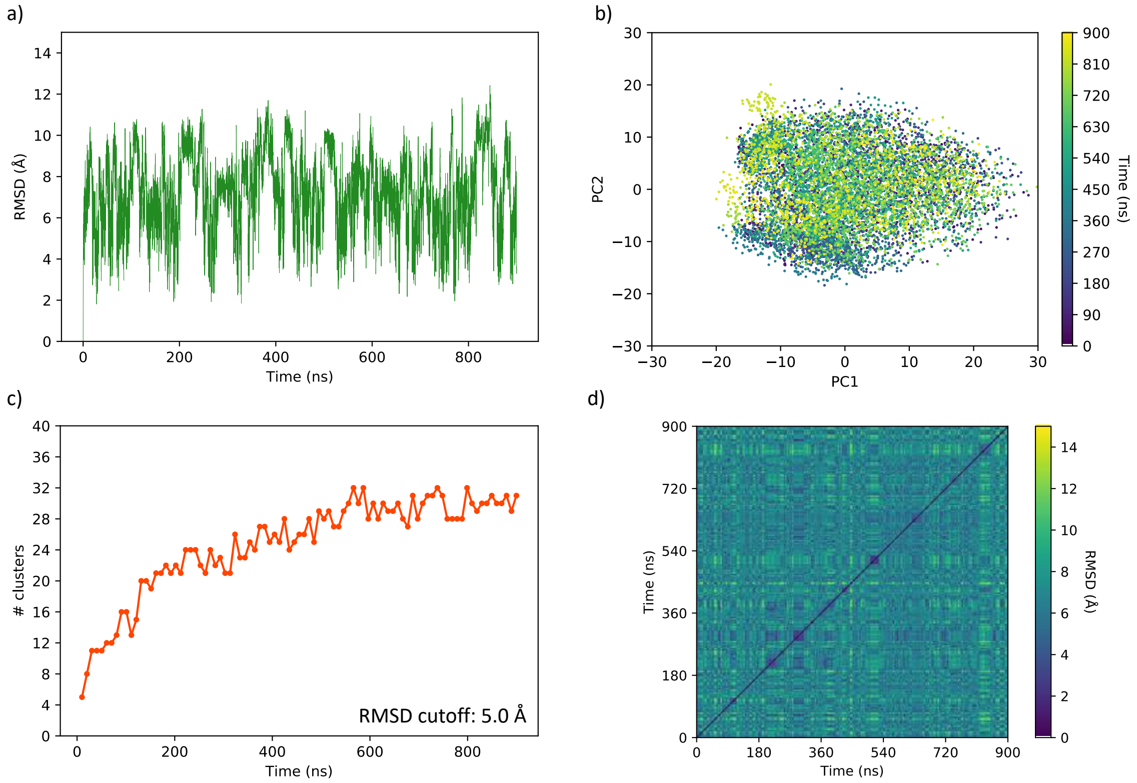

Supplementary Figure S1. Convergence studies for the MD simulation of peptide 8. (**a**) RMSD against the first frame of the trajectory. (**b**) Principal component analysis. Plot of the first principal component against the second principal component along the MD. The time is represented in a color scheme, from purple to yellow. (**c**) Cluster counting along the MD with a cluster cutoff of 5.0 Å. (**d**) All-to-all frames RMSD along the MD. The RMSD is represented in a color scale, from purple (lowest RMSD) to yellow (highest RMSD).

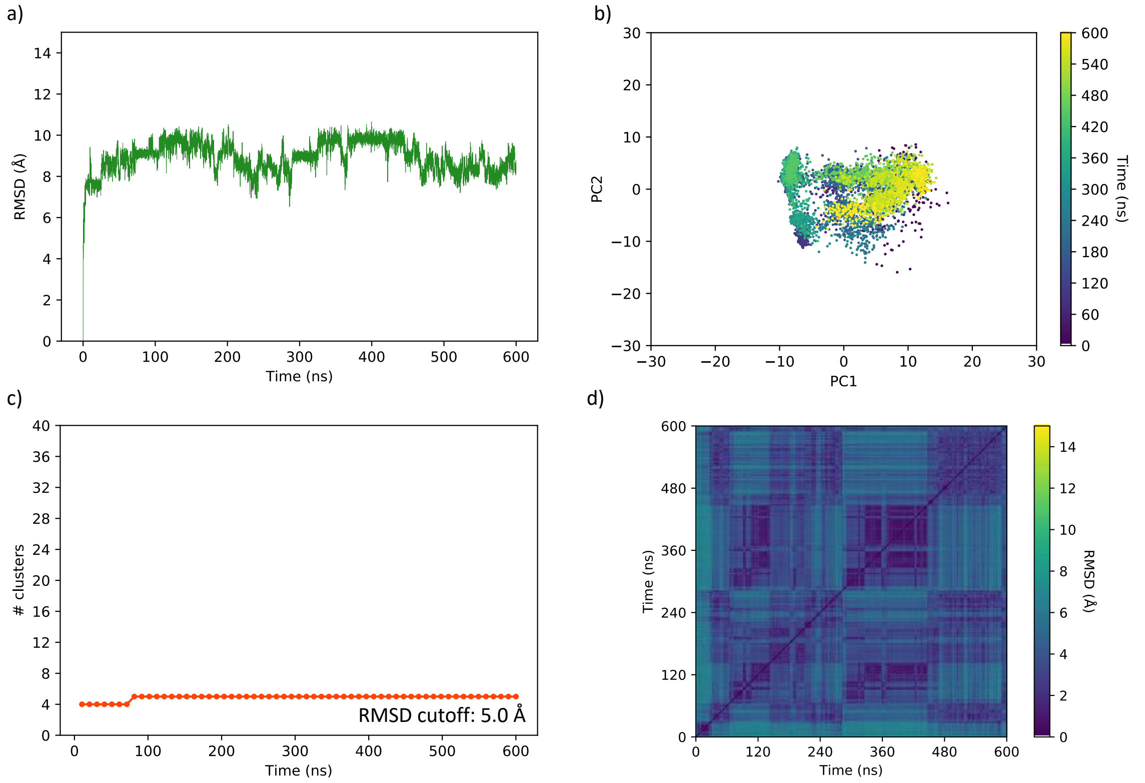

Supplementary Figure S2. Convergence studies for the MD simulation of CF-conjugate 11. (**a**) RMSD against the first frame of the trajectory. (**b**) Principal component analysis. Plot of the first principal component against the second principal component along the MD. The time is represented in a color scheme, from purple to yellow. (**c**) Cluster counting along the MD with a cluster cutoff of 5.0 Å. (**d**) All-to-all frames RMSD along the MD. The RMSD is represented in a color scale, from purple (lowest RMSD) to yellow (highest RMSD).

References

1. Martín-Vilà, M.; Muray, E.; Aguado, G.; Álvarez-Larena, A.; Branchadell, V.; Minguillón, C.; Giralt, E.; Ortuño, R. M. Enantioselective synthetic approaches to cyclopropane and cyclobutane β-amino acids. Synthesis and structural study of a conformationally constrained β-peptide. *Tetrahedron:Asymmetry* **2000**, *11*, 3569–3584.
2. Illa, O.; Olivares, J.-L.; Gaztelumendi, N.; Martínez-Castro, L.; Ospina, J.; Abengozar, M.-A.; Sciortino, G.; Maréchal, J.-D.; Nogués, C.; Royo, M.; Rivas, L.; Ortuño, R.M. Chiral cyclobutane-containing cell-penetrating peptides as selective vectors for anti-Leishmania drug delivery Systems. *Int. J. Mol. Sci.* **2020**, *21*, 7502.
3. Choi, S.; Isaacs, A.; Clements, D.; Liu, D.; Kim, H.; Scott, R. W.; Winkler, J. D.; DeGrado, W. F. De novo design and in vivo activity of conformationally restrained antimicrobial arylamide foldamers. *Proc. Natl. Acad. Sci. USA* **2009**, *106*, 6968–6973.
